# Supplementary material for: Microcavity phonoritons – a coherent optical-to-microwave interface
Source: Nat Commun. 2023 Sep 18;14:5470. doi: 10.1038/s41467-023-40894-7 (PMC10507037; doi:10.1038/s41467-023-40894-7)
Supplement: Supplementary file 1 — Supplementary Information [file 41467_2023_40894_MOESM1_ESM.pdf]

Supplementary Material: Microcavity phonoritons – a coherent optical-to-microwave interface

Alexander Sergeevich Kuznetsov*,[1] Klaus Biermann,[1] Andres Alejandro Reynoso,[2,3,4] Alejandro Fainstein,[2,3] and Paulo Ventura Santos[1]

[1] *Paul Drude Institute for Solid State Electronics, Leibniz Institute in the Research Association Berlin e. V., Hausvogteiplatz 5-7, 10117 Berlin, Germany* *

[2] *Bariloche Atomic Centre and Balseiro Institute, National Council for Scientific and Technical Research, 8400 S.C. de Bariloche, R.N., Argentina*

[3] *Institute of Nanoscience and Nanotechnology, National Council for Scientific and Technical Research,8400 Bariloche, Argentina*

[4] *Department of Applied Physics II, University of Seville, E-41012 Sevilla, Spain*

I. Polaromechanical microcavities

A. Detailed structure

The structured polariton microcavity (MC), which is displayed in Fig. 1 of the main text, was grown by molecular beam epitaxy (MBE) on a nominally intrinsic, double-side polished GaAs (001) wafers (Wafer Technology Ltd.). The layer structure was designed to simultaneously confine photons with an optical wavelength λ (defined as the ratio $\lambda = \lambda_L/n_s$ between the free space wavelength $\lambda_L = 810$ nm and the effective refractive index, n_s, of the MC spacer) and acoustic phonons with wavelengths λ and 3λ [1]. Phonons with these wavelengths will be referred to as λ-phonons and 3λ-phonons, respectively. For longitudinal acoustic (LA) phonons propagating along the $z||\langle 001\rangle$ direction of GaAs, these wavelengths correspond to phonon frequencies of approx. 21 and 7 GHz, respectively. The layer structure of the sample is summarized in Supplementary Table 1. The spacer region of the MC has an optical thickness $3\lambda/2$ (corresponding to half the LA phonon wavelength) and includes six 15-nm thick GaAs quantum wells (QWs) placed close to the antinodes of the optical field and the acoustic field.

The spacer is sandwiched between a lower (LDBR) and an upper (UDBR) distributed Bragg reflectors (DBRs). Each DBR period consists of a stack of three pairs of $\mathrm{Al_{x_1}Ga_{(1-x_1)}As/Al_{x_2}Ga_{(1-x_2)}As}$ layers, each with an optical thickness $\lambda/4$. The thicknesses d_1 and d_2 and Al compositions x_1 and x_2 of the layers pairs are listed in Supplementary Table 1. This configuration yields a strong modulation of the optical and acoustic properties with periodicities of $\lambda/2$ and $3\lambda/2$, which enables the simultaneous reflection of both photons with optical wavelength λ and phonons with wavelengths λ and 3λ.

Supplementary Table 1: Layer structure of the microcavity samples. The DBRs consist of stacks of $\mathrm{Al}_{x_1}\mathrm{Ga}_{1-x_1}\mathrm{As}$ and $\mathrm{Al}_{x_2}\mathrm{Ga}_{1-x_2}\mathrm{As}$ layers with thicknesses d_1 and d_2 and Al compositions x_1 and x_2, respectively. d_T and n_{rep} are, respectively, the total thickness and the number of periods.

Region	d_T (nm)	n_{rep}	$d_1 : d_2$ (nm)	$x_1 : x_2$	Comment
UDBR	4155	11	58.12:63.14	0.10:0.50	pair_1
			58.12:67.63	0.10:0.90	pair_2
			63.14:67.63	0.50:0.90	pair_3
growth interruption	—	—	—	—	
spacer	289	1	289	0.10	including 6 GaAs QWs
LDBR	5289	14	63.14:67.63	0.50:0.90	pair_3
			58.12:67.63	0.10:0.90	pair_2
			58.12:63.14	0.50:0.10	pair_1
substrate					

The intracavity traps are created by interrupting the MBE growth after the deposition of the upper part of the spacer layer (i.e., after the growth of the QWs)[2]. The sample was then extracted from the growth chamber and its surface was photolithographically patterned by shallow (17 nm) etching. It was subsequently reintroduced into the growth chamber for the overgrowth of the UDBR. Due to the conformal nature of the MBE growth, the photolithographically

* Corresponding author, e-mail: kuznetsov@pdi-berlin.de

imprinted profile is maintained during the overgrowth, leading to the formation of a thicker spacer region at the mesa sites (denoted here as the non-etched regions) in-between etched areas. The lateral trap confinement results from the lower acoustic and optical cavity resonance energies at these positions. The overgrowth was carried out at a lower deposition temperature (420°C as compared to the temperature of 640°C employed for the first growth cycle) to minimize the smoothing of the lateral interfaces due to surface diffusion of impinging atoms.

B. Optical and acoustic response

The reflection and transmission of optical and acoustic waves through the etched and non-etched regions of the sample were determined using a transfer matrix procedure to calculate the optical and acoustic fields following the excitation (i.e., optical or acoustic) at the sample surface. The results are summarized for the optical and acoustic modes in the left and right panels for Fig. 1.

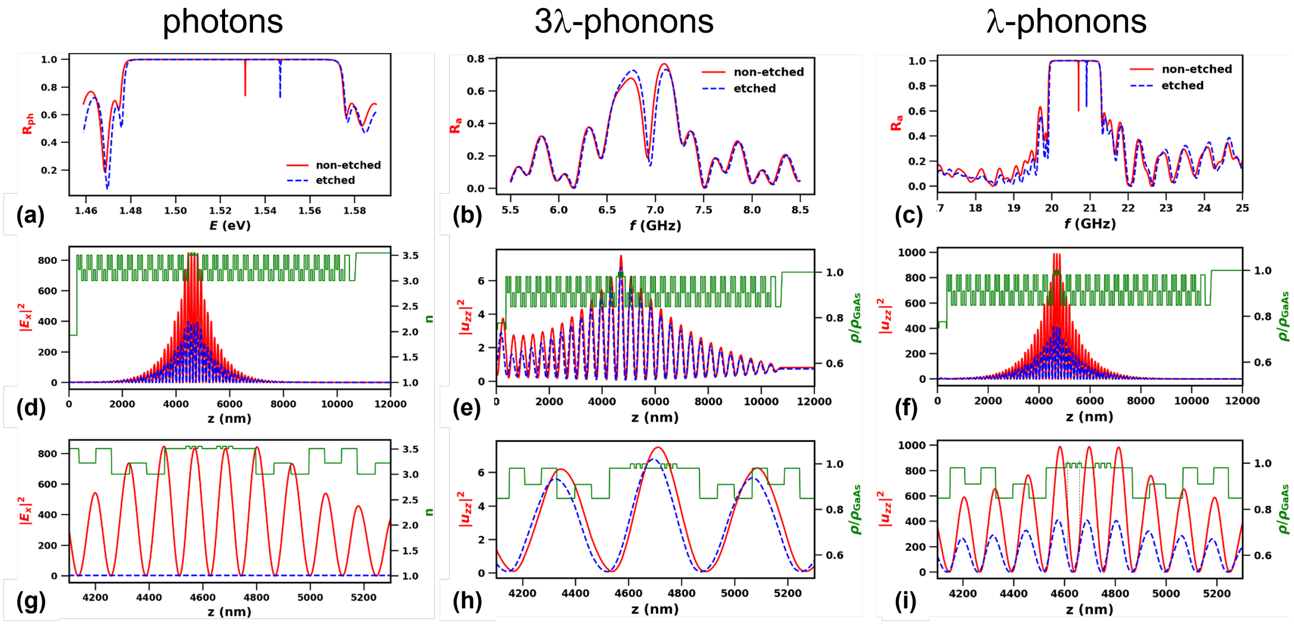

Fig. SM 1: **Optical properties of the structured microcavity.** (a-c) Calculated optical (R_{ph}) reflectivity and acoustic reflectivity (R_a) for the longitudinal acoustic (LA) phonons with wavelengths 3λ and λ, respectively. The dashed blue and solid red curves are for the etched and non-etched areas of the sample. The dips within the stop-band of high reflectivity between 1.48 and 1.57 eV in (a) 6.6 and 7.3 GHz in (b), and between 20 and 21.5 GHz in (b) and (c) are, respectively, for the polariton, and phonon modes confined within the MC spacer. (d-f) Depth dependence (z direction) of the squared electric $[E^2(z)]$ and strain $(|u_{zz}^2(z)|)$ fields at the reflectivity minima within the stop-bands in (a-c). (g-i) Close-up around the MC spacer area of the profiles in (d-f) within the MC spacer. The green curves in (d-g) display the depth dependence of the refractive index (n) of the layers. The green curve in (e-f) and (h-i) displays the corresponding depth dependence for the density ρ (normalized to the GaAs density, ρ_{GaAs}).

Figure 1(a) displays the optical reflectivity spectrum R_{ph} for photons impinging at normal incidence on the sample surface. The calculations of the optical response do not include the effects of the excitonic resonances: they yield, therefore, only the bare optical MC mode (C). The blue and red curves apply for the etched and non-etched regions of the sample. The high reflectivity band between 1.48 and 1.57 eV is the photon stop-band introduced by the MC DBRs. The sharp dip is the photon cavity mode C, which in the etched areas (blue curve) is blue-shifted by 16 meV with respect to the non-etched ones (red curve). The dependence of the optical field for the C mode is illustrated in Fig. 1(d) and in the close-up around the spacer region of Fig. 1(g). Here, the vertical axis displays the squared amplitude of the field $|E(z)|^2$ normalized to the one of the impinging optical beam. The green plots reproduce the depth dependence of the refractive index (n) in the MC structure. Here, one can clearly see the $\lambda/2$ and $3\lambda/2$ sequence of DBR layers required for simultaneous photon and phonon reflection. Note that the two sets of three QWs within the spacer are located around antinodes of the optical field, thus ensuring a strong overlap with the QW excitonic resonances.

The previous picture for photons also applies to phonons. The central and left panels of Fig. 1 show the acoustic reflectivities of the longitudinal acoustic modes with wavelenghts 3λ and λ, respectively. The DBRs introduce acoustic

stop-bands in the phonon reflectivity (R_a) spectrum [Fig. 1(b-c)] extending from 6.6 to 7.3 GHz and between 20 and 21.5 GHz for the 3λ and λ modes, respectively. The dips within the stop-bands are associated with the excitation of modes confined within the MC spacer. The reduced spacer thickness in the nERs blue-shifts the phonon frequency by approximately 27 MHz for the 3λ-phonons and by $\sim$ 100 MHz for the λ-phonons. The profiles for the squared strain field [Figs. 1(e-f)] are again confined within the spacer: the penetration length into the DBRs is much larger for the 3λ phonons than for the λ ones, where this penetration is comparable to the one for photons in Figs. 1(d). The longer penetration is due to the lower number of effective DBR stacks with $3\lambda/2$ periodicity: as a result, the acoustic quality factor for 3λ phonons of $Q_a \sim 200$ is much smaller than for photons and for λ-phonons.

The higher Q_a for the λ-phonons leads to much narrower resonances in Figs. 1(b-c). Despite the fact that the overlap of the strain field and the QWs has not been optimized for λ phonons, their average squared strain field over the QWs exceeds by approximately two orders of magnitude the one for the 3λ-modes cf. Figs. 1(e-f)]. The stronger coupling to excitons resulting from the higher amplitudes of the λ phonons has important consequences for self-oscillation effects. As will be discussed later, the phonon backreflection at the sample surfaces considerably increases the quality factors for both types of phonons[1].

II. Photoluminescence of confined polaritons

A. Light-matter coupling

Variations of the MBE fluxes along the sample surface create a slight reduction of the thickness of the MBE layers as one move from the center to the border of the substrate wafer. The thickness reduction blue-shifts the optical mode of the MC, while the excitonic resonances remain approximately constant. This relative variation between the optical and excitonic energies enables a precise determination of the Rabi (light-matter) coupling at different positions on the sample surface.

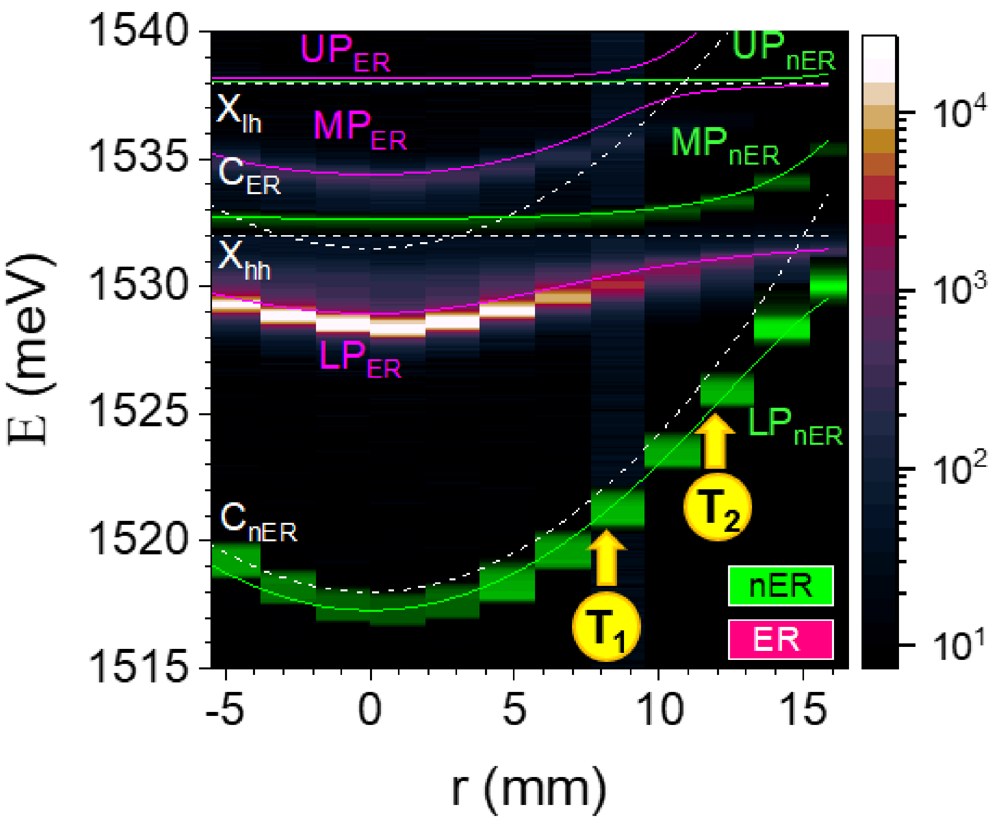

Fig. SM 2: **Spatial photoluminescence of the microcavity.** Photoluminescence lines recorded on non-etched (nER, green) and etched (ER, magenta) regions on the 2-inch MC wafer at different radial positions (r) relative to the wafer center ($r = 0$). The dashed lines show a fit to a photon-exciton coupled oscillator model for the coupling between the optical mode as well as the hh and lh excitons, yielding the three polariton modes LP (lower polariton), MP (middle polariton), and UP (upper polariton). The dashed lines show the bare photon, hh and lh exciton energies. Note that the former is blue-shifted in the ER regions. T_1 and T_2 mark the position of the intracavity traps discussed in the main text.

Figure 2 displays the energy of the polariton photoluminescence (PL) recorded on non-etched (green) and etched (magenta) regions as the function of the radial position on the sample (with r = 0 being the wafer center). At each position, one detects PL from the lower (UP), middle (MP), and upper (UP) polariton branches resulting from the coupling between the optical MC mode (C) with the electron heavy-hole (X_{hh}) and electron light-hole (X_{hh}) excitons in the QWs.

Supplementary Table 2: Parameters determined from fits of the spatial dispersion of the polaritons in extended etched (ER) and non-etched (nER) cavity regions.

Parameter	Value	Comment
E_{hh}	1532 meV	Bare heavy-hole exciton energy
E_{lh}	1538 meV	Bare light-hole exciton energy
C_{nER}	1518 meV	Bare cavity mode energy in non-etched region at $r = 0$ mm
C_{ER}	1531.5 meV	Bare cavity mode energy in etched region at $r = 0$ mm
$\Omega_{X_{hh}}$	6.0 ± 0.25 meV	Rabi-splitting energy for cavity-heavy-hole (for both regions)
$\Omega_{X_{lh}}$	2.0 ± 0.25 meV	Rabi-splitting energy for cavity-light-hole (for both regions)
m_{eff}	4.8×10^{-5}	Polariton effective mass in free electron mass

The solid lines are fits to a photon-exciton coupled oscillator model for the interaction between the three modes. The model assumes that the reduced spacer thickness blue-shifts the optical modes in the etched regions relative to the non-etched ones, while the excitonic energies remain constant. The fits yields three polariton modes LP, MP, and UP. The parameters used for the fits are summarized in Supplementary Table 2. The dashed lines show the fitted bare X_{hh} and X_{lh} exciton energies, as well as the radial-dependence of the photon energy (C) in the non-etched regions. LP is highly photonic at the center of the wafer ($r = 0$). The zero detuning between the C and X_{hh} energies is reached at $r = 15$ mm. Note that the lateral confinement potential for the intracavity traps, which is equal to the difference between the UP energies in the etched and non-etched region, reduces with r. T_1 and T_2 mark the position of the intracavity traps investigated here.

B. Polariton confinement and condensation

Figure 3a shows an exemplary PL map recorded along the spatial [-1-10] axis of a 4×4 μm^2 trap T_1. The measurement were carried out at 10 K under non-resonant optical excitation below the condensation threshold. Several confined levels can be easily identified. The spatially integrated spectrum is shown in the middle-section. Each level has a characteristic spatial profile of intensity, which reflects the squared profiles of the polariton wavefunctions. The spectrum can be faithfully reproduced using numerical simulations [2]. The simulated 2D spatial profiles are shown in the right-section.

Figure 3b presents the dependence of the trap spectrum on the excitation power of the non-resonant laser. The upper-section shows the integrated intensity of the ground-state (GS). Around ~ 50 mW power (dashed vertical line), polaritons transition to the polariton Bose-Einstein condensate (BEC). In the color map, the dashed horizontal line (labelled E_C) denotes the energy of the bare cavity mode that was used to fit the spatial spectrum of the trap. The energy difference between the E_C and the condensate in the GS indicates that above the condensation threshold the system is still in the strong-coupling regime.

C. Estimation of the polariton population

We estimate the optically excited polariton population in a trap as $N_{MP} = (1 - R) r_A \eta \frac{P_{Exc}}{\hbar\omega_{Exc}} \tau$. In the latter expression, R is the MC reflectivity at the excitation wavelength, r_A is the ratio between the trap and the excitation spot area, η is the fraction of excited electron-hole pairs that are stimulated into the BEC from the exciton reservoir, P_{Exc} and $\hbar\omega_{Exc}$ are the excitation power and photon energy, and τ is the exciton reservoir lifetime, which is estimated to be on the order of $\tau = 1$ ns. Assuming R = 0.5 for the excitation wavelength of 760 nm, $r_A = 0.01$ for a 4×4 μm^2 trap and a Gaussian excitation spot with the radius 20 μm, and $\eta = 0.1$, we obtain $N_{MP} = 2400 \times P_{Exc}$ for P_{Exc} in mW. Hence, at the condensation threshold $P_{Exc} = 50$ mW (cf. Fig. 3), we deduce $N_{MP} \approx 10^5$ for the BEC population.

D. Acoustic frequency comb in PL of confined polaritons

Figure 4c shows the dependence of the PL spectrum of a trap below the condensation threshold on the radio frequency (F_{RF}) applied to the transducer. The spectra were recorded for a fixed RF power. The displayed F_{RF} range corresponds to the range of the 3λ acoustic mode of the MC. The data shows pronounced energy-modulation of all confined levels, which appears as a comb of narrow diamond-like shapes. Phonons escaping from the spacer region

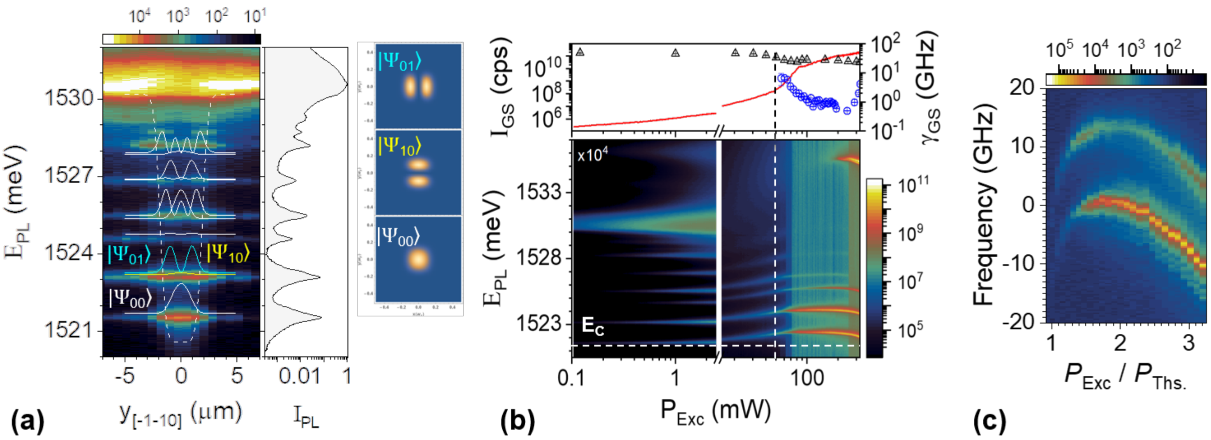

Fig. SM 3: **Photoluminescence of confined polaritons.** (a) The map of PL of a $4 \times 4\mu\ m^2$ trap resolved in energy and in space measured at 10K. Several confined polariton modes are visible below the barrier energy around 1530 meV. The dashed and solid white lines on top of the data are the simulated cross-section of the polariton confinement potential and squared wavefunctions, respectively. The section on the right shows a spatially integrated PL spectrum of the trap. The three sections further to the right display calculated spatial profiles of the square wavefunctions for the three lowest energy levels. (b) Lower part: a map showing trap PL spectrum as the function of the optical excitation power (P_{Exc}). Upper part: total integrated PL as the function of the excitation power (solid line) and the linewidth (γ_{GS}) of the trap ground state (GS). The black triangles are values measured with a single grating spectrometer, while the blue circles are measurements using the FP etalon. The vertical dashed line designates the condensation threshold power ($P_{\text{Th.}} \approx 60$ mW). The horizontal dashed line depicts the expected energy of the bare cavity mode (E_{C}). (c) As-measured spectrum of the trap T_1 GS as the function of the excitation power (P_{Exc}) normalized to the threshold power

can be re-fed to this region via acoustic reflection at the polished sample surfaces. As a consequence, the acoustic response of the MC is given by a combination of the relatively low quality ($Q_{MC} \approx 180$) MC mode and the higher quality ($Q_{comb} > 5000$) Fabry-Perot modes of the cavity formed by the whole sample thickness [1]. The modulation amplitude increases for the higher confined levels, which have a higher polariton excitonic content: this behavior is consistent with a modulation mechanism dominated by the deformation potential interaction. The envelope of the modulation amplitude gives the shape and the width of the acoustic mode of the MC (localized within the spacer).

E. Population of rf-generated phonons

First, we determine the rf-induced strain from the high-resolution PL spectrum. Specifically, Fig. 2(a) of the main text shows ± 4 phonon sidebands for an rf amplitude of $P_{RF}^{0.5} = 0.14\ W^{0.5}$. This corresponds to the modulation amplitude of $\Delta E = 4 \times \hbar\Omega_M \approx 114\ \mu$eV for the phonon frequency of $\Omega_M = 2\pi \times 7$ GHz. The value of the zz-component of the strain corresponding to ΔE can be found using $\epsilon_{zz} \approx \Delta E/(X^2 \times a_h)$ (cf. Eq. 19 and neglecting the small contribution associated with the deformation potential. For the lower polariton Hopfield coefficient $X^2 = 0.08$ and GaAs hydrostatic deformation potential a_h from cf. Supplementary Table 3, we obtain $\epsilon_{zz} = 1.6 \times 10^{-4}$.

The phonon population in a $4 \times 4\mu\ m^2$ trap can be determined as $N_{phon} = \epsilon_{zz}/u_{zz}^{ZPM}$, where $u_{zz}^{ZPM}\epsilon_0 = 1 \times 10^{-8}$ – is the strain of a single 7 GHz phonon, determined from a numerical simulation described in SM Sec. V B (cf. Fig. 12). Thus, we estimate phonon population $N_{phon} = 16000$ for $P_{RF}^{0.5} = 0.14\ W^{0.5}$.

III. High-resolution spectroscopy of polariton condensates

A. Optical setup

Figure 5 sketches a high-resolution optical setup used to detect phonon sidebands in the emission of polariton BECs. The sample is mounted in an optical Helium-flow cryostat with rf-connections for the excitation of the BAWRs. The pump laser and control laser beams are used to excite the sample. A part of the collected PL is diverted using a mirror and coupled into a single mode (5 μm core diameter) fiber. A long-pass filter blocks the scattered light from the pump laser. The fiber guides the PL to a piezo-tunable Fabry-Perot etalon (FP). The FP has a finesse of ~ 240 and free spectral range (FSR) of 68 GHz. The transmission wavelength of the FP is tuned by an external voltage (V_{piezo}) source, controlled from a PC. The PL signal filtered by the FP is guided by another single-mode fiber to the entrance of a single grating spectrometer. The spectrometer resolves the PL from the different FSRs of the FP,

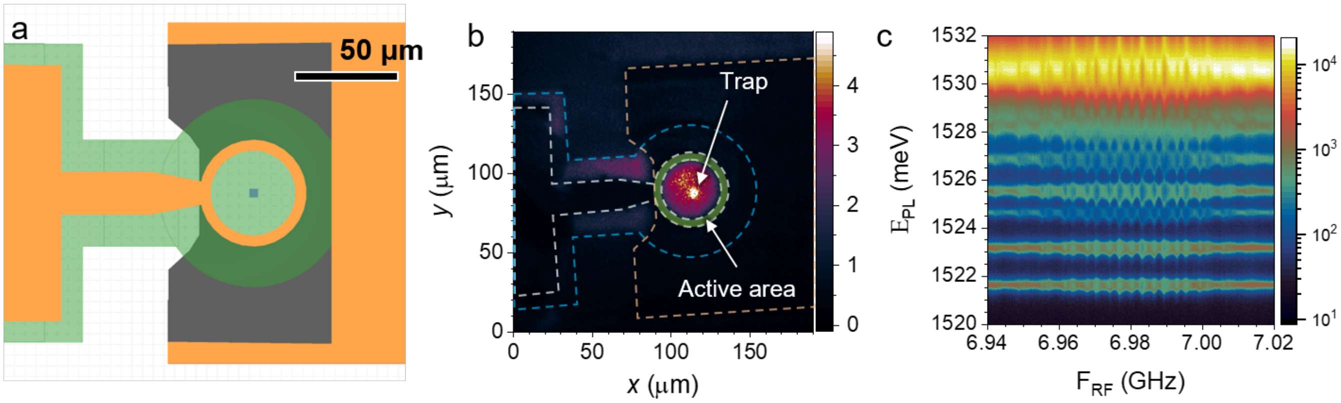

Fig. SM 4: **Acoustic modulation of confined polaritons.** (a) Schematic top view of the sample. The large features are the BAWR top contact (orange color), bottom contact (dark color) and ZnO layer (semitransparent green). Phonons are generated in the circular area that corresponds to the spatial overlap of the bottom and top contacts. A small square at the center of the circular aperture is the polariton trap. (b) Spatially resolved (energy-integrated) photoluminescence (PL) image map showing the bright emission of trap T_2. The superimposed dashed lines are outlines of the BAWR electrodes and its active area. c. Acoustic frequency comb in PL of confined polaritons. Dependence of the PL spectrum (below the condensation threshold) of a 4×4 μm^2 trap on the RF frequency (F_{RF}) applied to a BAWR transducer. The F_{RF} range corresponds to the range of the MC acoustic mode.

which is then detected by a nitrogen-cooled CCD. A custom-made software was used to control the voltage applied to the FP, which allowed to conduct scans with a resolution down to 0.28 GHz. In order to avoid temperature induced drifts, the FP was actively stabilized with an external heater. In this configuration, we loose the spatial information. In order to avoid collecting PL from other traps on the sample, we carried out our measurements on the sample area containing an isolated trap.

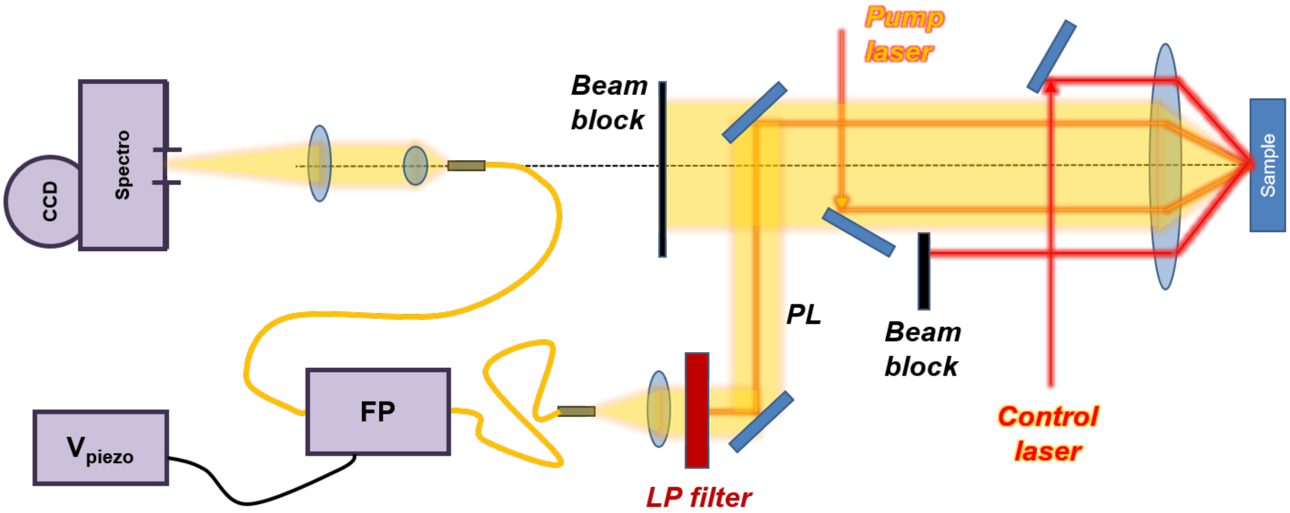

Fig. SM 5: **Setup for phonon sideband spectroscopy of condensates.** A sketch of the experimental setup for high-resolution optical spectroscopy of phonon sidebands in the emission of confined polariton condensates. The sample is mounted in a liquid He cryostat with rf connection to drive the BAWRs. The orange and red arrows show the optical paths of the pump and control laser beams, respectively, used to excite the sample. The PL signal (represented by the yellow area) is fiber-coupled to a Fabry-Perot etalon (FP) tunable by a piezo-controller (V_{piezo}). The output of the FP is directed via a second fiber to a spectrometer with a CCD detector.

B. Polariton BEC linewidth

Figure 3(c) displays a high-resolution PL spectrum of the trap T_1 ground state (GS) as the function of the pump laser power above the condensation threshold. The trap GS is split into two levels (see main text for details), separated by $2 \times \hbar\Omega_M$, where $\hbar\Omega_M$ is the phonon energy with $\Omega_M/2\pi = 7$ GHz. The initial blueshift is due to the polariton-polariton interactions, while above ~ 120 mW the modes redshift due to the optically-induced heating.

The upper-section of the Fig. 3(b) shows the full-width at half-maximum ($\gamma_{\rm GS}$) of the trap GS. The black triangles were determined from a low-resolution measurement (without the FP etalon and resolution of $\sim$ 25 GHz). These measurements yield $\gamma_{\rm GS} \sim 50$ GHz below the condensation threshold. This value reduces to the resolution limit of $\sim$ 20 GHz above the threshold. The blue circles give values of $\gamma_{\rm GS}$ for the lower energy peak in Fig. 3(c) measured with the high-resolution (of $\sim$ 0.3 GHz) using the piezo-tunable FP etalon. In the power range of 90–200 mW the linewidth is $\sim$ 1 GHz, which corresponds to a coherence time of $\sim$ 1 ns. Around 250 mW, the linewidth reaches its minimum of $\sim$ 0.5 GHz (coherence time of 2 ns). The increase of the linewidth above 250 mW is attributed to optically induced heating.

C. Self-induced sideband modulation

The color coded plot of Fig. 6(a) displays as-measured high-resolution spectra of the condensate emission of the 4×4 μm^2 trap as a function of the optical excitation power, $P_{\rm exc}$. These high-resolution measurements were recorded using the FP setup described above. The energy scale is specified in terms of the voltage applied to the piezo controlling the etalon. The figure shows a series of sidebands around the main emission line – the zero-phonon line (ZPL, marked by a red arrow). The spectroscopic features repeat with a periodicity determined by the free spectral range (FSR) of the etalon. All lines experience a small blueshift (less that the FSR) with increasing $P_{\rm exc}$, which is attributed to polariton-polariton interactions. Figure 6(b) displays the same data after artificially aligning the ZPL of all the curves.

The same measurement also captures the emission of the excited state (ES) of the trap, as illustrated in Fig. 6(c). For this particular detuning, the condensation threshold for the ES is $\sim$ 4 times higher than the threshold for the GS in Fig. 6(b). Both the GS and ES spectra show a series of week sidebands around the ZPL. The origin of these lines is discussed in the main text.

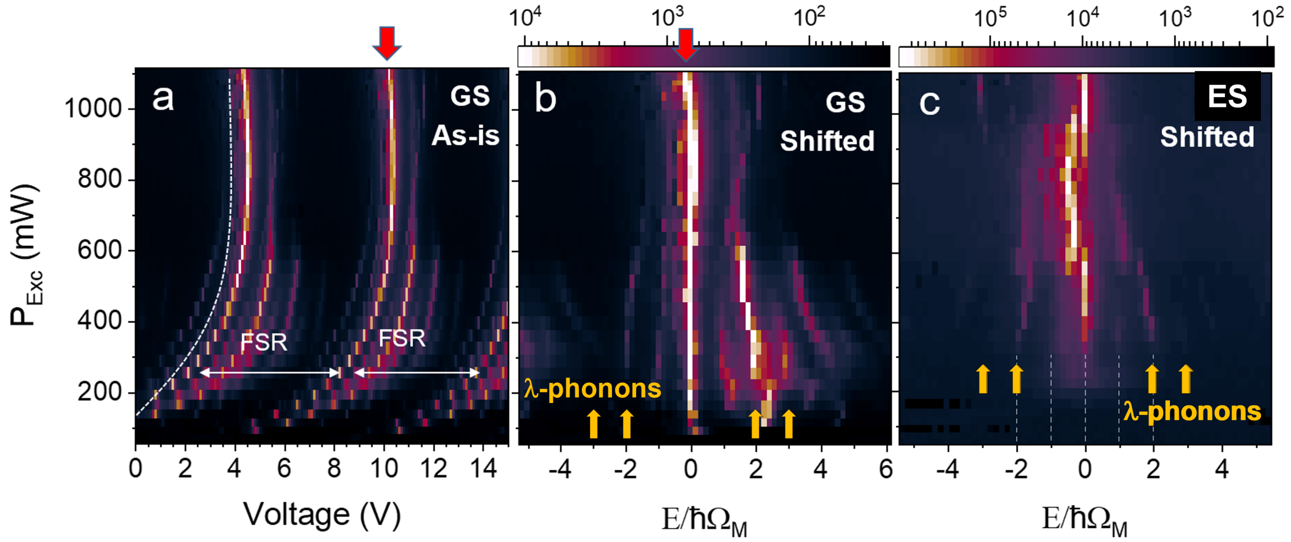

Fig. SM 6: **Self-induced sidebands**. (a) Raw maps of the ground state (GS) PL recorded for increasing optical excitation power $P_{\rm exc}$ focused on a spot of 10 μm positioned over a 4×4 μm^2 trap. The energy (horizontal axis) is specified in terms of the voltage applied to the piezo controlling the etalon with a voltage to frequency conversion factor of 6V per free spectral range of 68 GHz. The zero-phonon line (ZPL) is marked by a red arrow. (b) Same data after aligning all of the ZPL. The scale is stated in units of the LA 3λ phonon frequency $\hbar\Omega_{\rm M}$. (c) Shifted data for the excited state of the trap. The yellow arrows display the energies of the TA and LA λ-phonons.

IV. RF-induced Sidebands

A. Raw sidebands data and correction

Figure 7(a) shows as measured high-resolution spectra of a trap GS as the function of RF power (P_{RF}^2) and fixed $F_{RF} \approx 7$ GHz. The imperfect transduction of RF power into acoustic leads to local heating of the sample. The latter is the origin of the redshift at higher P_{RF}^2. To simplify the analysis and presentation each spectrum was shifted horizontally by a small amount in order to match the energies of the zero-phonon line, cf. Fig. 7(b).

B. Bessel-function fitting

Figure 8(a) displays a PL spectral map of the first excited state of a trap recorded for different amplitudes of the bulk acoustic wave (BAW) $A_{\mathrm{BAW}} = \sqrt{P_{\mathrm{rf}}}$. Here, P_{rf} is the nominal rf power applied to the BAWR. Individual PL spectra for different acoustic amplitudes are displayed by the symbols in the central panel. With increasing BAW amplitudes, one observes the appearance of an increasing number of well-defined sidebands displaced by multiples of the BAW quantum, hf_{BAW}.

In the presence of a coherent harmonic driving of the BEC, the PL spectrum is expected to be proportional to $P[\omega]$:[3–5]

$$P[\omega] = \sum_{n=-\infty}^{\infty} \frac{\Gamma}{2\pi} \frac{J_n^2(\chi)}{[\omega - (\omega_0 - n\,\omega_a)]^2 + (\frac{\Gamma}{2})^2}, \tag{1}$$

that is, a sum of Lorentzians with linewidths Γ, weighted by squared Bessel functions $J_n^2(\chi)$. χ is the dimensionless amplitude modulation of the harmonic drive ω_0, i.e., $\chi = \Delta\omega_0/\omega_a$, where ω_a is the driving frequency. The Lorentzians have maxima at frequencies $\omega = \omega_0 - n\omega_a$, where n is an integer. Note that since $\sum_{n=-\infty}^{\infty} J_n^2(\chi) = 1$, $\int_{-\infty}^{\infty} P[\omega]dw = 1$ (see [functions.wolfram.com/Bessel-TypeFunctions/BesselJ/23/01/]), the effect of the modulation in Eq. 1 is thus to distribute the oscillator strength among the sidebands without affecting the overall PL intensity.

The solid lines in Figure 8(c,d,e) are fits of Eq. 1 to the experimental data. The latter yield the modulation amplitudes χ and linewidth Γ displayed as a function of A_{BAW} in Fig. 8(f) and (g), respectively. χ is proportional to A_{BAW}.

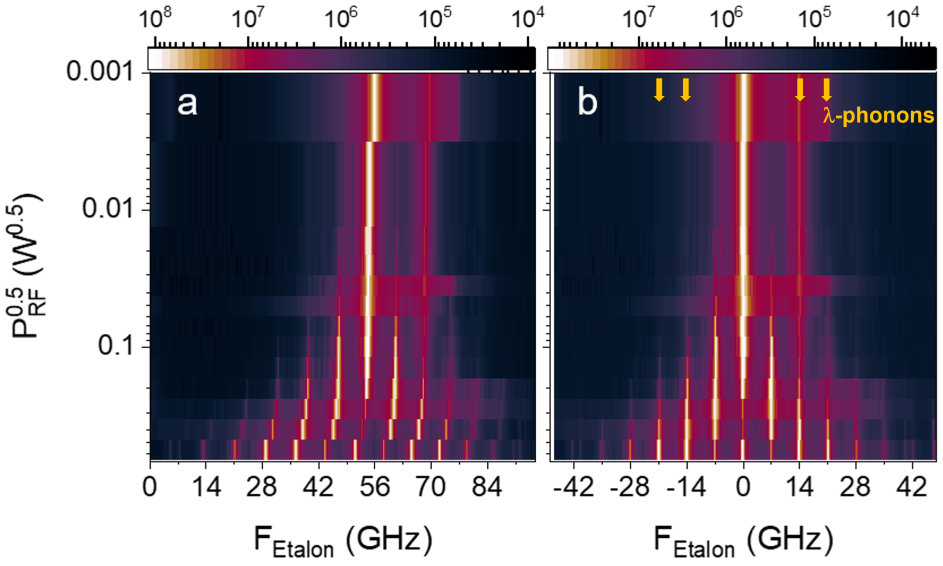

Fig. SM 7: **RF sidebands.** Dependence on the RF power (P_{RF}^2) applied to the BAWR of the high-resolution PL spectrum of the polariton ground state (a) as-measured and (b) after energy correction to match the zero phonon line for the different P_{RF}^2. The yellow arrows display the energies of the TA and LA λ-phonons.

C. RF-induced sidebands in the excited state

The experimental curves for the first excited state of the trap, shown in Figs. 8(c,d,e), are fitted well with a sum of Lorentzians with linewidths δE, weighted by squared Bessel functions $J_n^2(\chi)$, where χ – is the modulation amplitude, as described above.

Figure 8g shows the dependence of the sideband linewidth on the normalized acoustic amplitude (A_{BAW}). Similarly to the GS (see Fig. 2g in the main text), the linewidth [$\delta E(A_{BAW})$] extracted from the fits decreases sharply by a factor of two from $\delta E(0.1) = 0.22\hbar\Omega_M = 1.75$ GHz to $\delta E(0.2) = 0.1\hbar\Omega_M = 0.8$ GHz and remains almost constant afterwards.

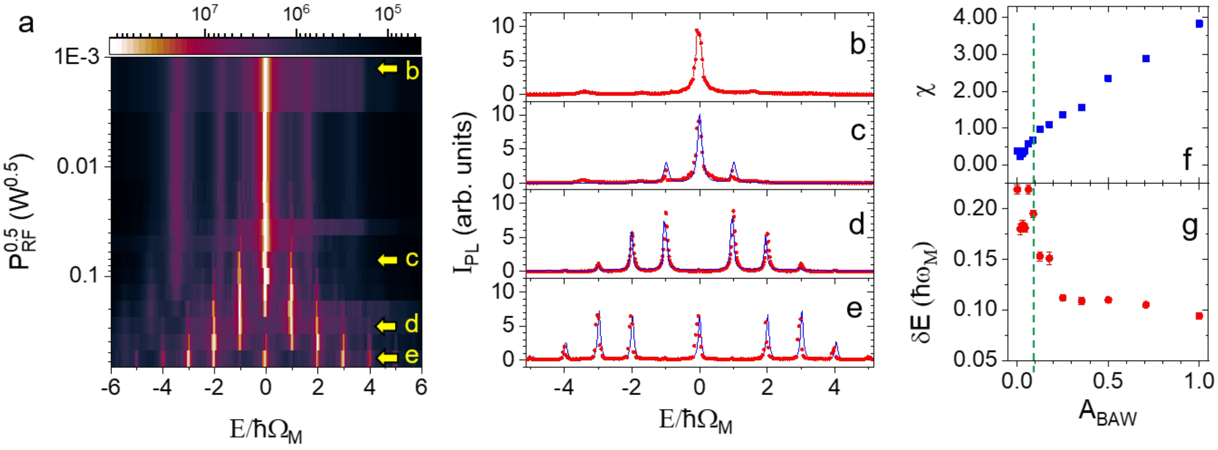

Fig. SM 8: **RF sidebands in excited state.** (a) Dependence of the first excited state emission spectrum of a trap on acoustic amplitude $\sim \sqrt{P_{RF}}$. (b-e) PL spectra corresponding to the $\sqrt{P_{RF}}$ values indicated by yellow arrows (and letters) in panel a. The red dots are the data points while the blue solid lines are fits to Eq. 1. Dependence of the fitted modulation amplitude (f) and the linewidth (g) on the normalized acoustic amplitude. The vertical dashed green line in (f) and (g) indicates the acoustic amplitude for which the first sidebands appear. The error bars in (g) correspond to the standard error of the Lorentz function used to fit the corresponding peaks.

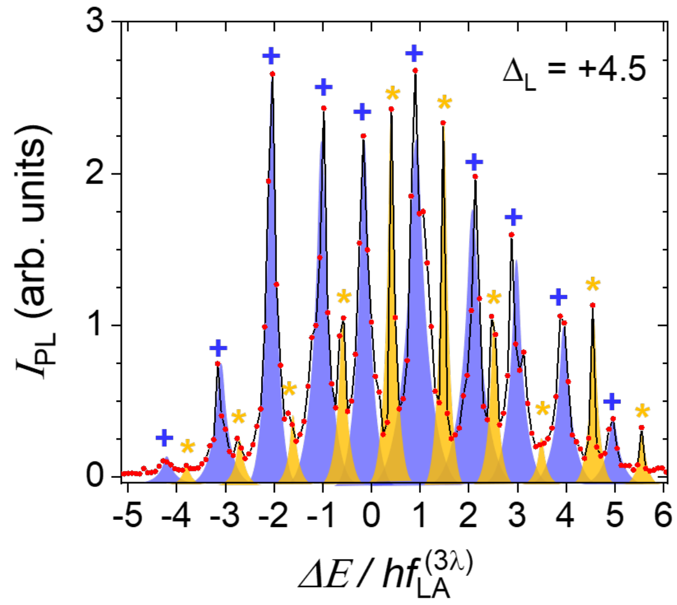

Fig. SM 9: **Polariton and laser combs.** Exemplary spectrum corresponding to the laser detuning of $\Delta_L/hf_{LA}^{(3\lambda)} = +4.5$ (cf. Fig. 4b in the main text) that shows two phonon-induced frequency combs: the one of the phonoriton and one of the resonant laser. The peaks of the phonoriton comb are designated by blue + symbols, while the peaks of the laser comb are indicated by orange * symbols. The blue and orange shapes are guides to the eye.

V. Theoretical Background

This section theoretically analyses the coupling between confined phonons and polaritons, which can lead to self-oscillations in an intracavity trap. We start by describing the confined polariton and phonon in the trap and then proceed to the calculations of the interaction between them mediated by the deformation potential mechanism.

Both polaritons and phonons are tightly confined within the traps, which is assumed to have a thickness $m_z\lambda_{\mathrm{BAW}}/2$, where λ_{BAW} is the acoustic wavelength of the 3λ LA BAW. In order to describe the polariton and phonon confined fields, we use a reference frame with the x, y, and z axes along the $[1\bar{1}0]$, [110], and [001] crystallographic directions, respectively. In the discussion to follow, a reference will also be made to the conventional cartesian frame with axes $x_c||[100]$, $y_c||[010]$, and $z_c||z||[001]$. Unless otherwise specified, the parameters used in the calculations are those listed in Supplementary Table 3.

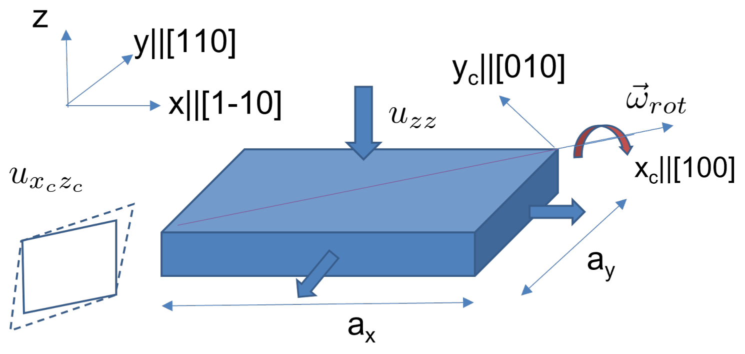

Fig. SM 10: **Hybrid trap confining phonons and polaritons.** The trap is assumed to have a rectangular shape with lateral dimensions $a_x = a + \Delta a/2$ and $a_y = a - \Delta a/2$, respectively, with $\Delta a \ll a$.

In order to enhance the optomechanical coupling, the QWs (with a thickness of 15 nm much smaller than λ_{BAW}) are placed close to an antinode of the light field, which is assumed to be at a position $z = 0$ [cf. Fig. 1(c)]. The MC was designed to ensure that the uniaxial strain along $z||[001]$ reaches its maximum amplitude $u_{zz,0}$ close to the same position [cf. Fig. 1(f)]. We will consider here only intra-cavity traps with a nominally square shape with the side length (a) in the x-y plane. The MBE growth dynamics, however, distorts the trap geometry [2]. As a consequence, the lateral sizes of the trap will be taken to be $a_x = a + \Delta a/2$ and $a_y = a - \Delta a/2$ along the x and y directions, respectively, as illustrated in Fig. 10. The small size difference $\Delta a \ll a$ takes into account the anisotropic nature of the MBE overgrowth process on a structured surface, which yields traps with different sizes even for the overgrowth on a perfectly square mesa [2]. The trap dimension along the z direction will be taken equal to the thickness of the MC spacer $d_s = m_z \frac{\lambda}{2n_s}$, where m_z is an odd integer and n_s is the average refractive index of the spacer layer.

A. Confined polariton modes

The envelope function of the confined polariton field in a trap can be written as:

$$\Psi^{(s)}_{(\mathrm{p_x p_y})}(x,y) = \sqrt{\frac{8k_xk_y}{3\pi^2}}\cos\left(k_xp_xx + \frac{1}{2}\pi(p_x-1)\right)\cos\left(k_yp_yy + \frac{1}{2}\pi(p_y-1)\right). \quad (2)$$

Here, $k_z = \pi/\lambda_{\mathrm{BAW}}$, $k_i = \pi/\ell_i$ ($i = x, y$), and the indices (p_i, p_j) (with $i = x, y$) are the number of the transverse lobes (i.e., perperdincular to z) of the polariton mode along the x and y directions, respectively. The previous equation applies for states with confinement energy shifts much smaller than the height of the trap potential barrier. The prefactor within the square root is a normalization factor ensuring that $|\langle\Psi^{(s)}_{(\mathrm{p_x p_y})}\rangle|^2 = 1$. As will be discussed in detail later, there are two polariton modes for each (p_xp_y) pair labeled by the pseudo-spin superscript index s.

For small asymmetries $\Delta a/a$, the energy of the (p_xp_y) polariton polariton state can be written as:

$$E^{(p_xp_y)}_{pol} = \frac{1}{3}(p_x^2 + p_y^2)E_{01} \quad (3)$$

where

$$E_{01} = \frac{3\hbar k_\perp^2}{2m_p}. \quad (4)$$

denotes the energy difference between the ground (GS) and the first excited state (ES) of the square potential. In the previous expression, $k_\perp = 2\pi/a$, and m_p the polariton mass.

1. *Confinement effects on excitonic states*

The high-resolution spectroscopic measurements on Fig. 2 (main text) shows a splitting of the polariton GS into two pseudo-spin levels (index s in Eq. 2). This splitting calls for a more detailed description of the coupling between excitonic and photonic levels confined in the lateral trap potential. In the case of bare excitons, while the electron states have a simple s-type electronic wavefunction, the valence band states are superpositions of p-like $\langle X\rangle$, $\langle Y\rangle$, and $\langle Z\rangle$ orbitals, which can be mixed by the lateral confinement potential. The $\langle X\rangle$ and $\langle Y\rangle$ orbitals are degenerate for a square trap. This degeneracy can be lifted by a difference Δa between the trap dimensions along the x and y directions according to:

$$\Delta_{\mathrm{XY}} = -2\left(\frac{\hbar^2 k_\perp^2}{2m_X}\right)\frac{\Delta a}{a} \approx -2\frac{m_{pol}}{m_X}E_{01}\frac{\Delta a}{a}, \tag{5}$$

where m_X is the effective excitonic mass. For the right-most term of this equation, the factor $\left(\frac{\hbar^2 k_\perp^2}{2m_X}\right)$ has been expressed in terms of the energy difference ΔE_{01} between the first excited and ground polariton states of the trap. Since $m_{pol} \ll m_X$, the lateral confinement shift of the exciton states can be neglected and Δ_{XY} assumed to vanish. Furthermore, we will neglect fine splitting effects related to the exciton spin levels based on the fact that the light-matter interaction considerably increases the energetic splitting between these dark and bright exciton levels.

2. *Confinement effects on the photonic states*

We now introduce the bare photonic states and their coupling to the excitons. The bare states will be described in a basis $\left(L_R = \frac{1}{\sqrt{2}}(L_{\mathrm{TM}} + iL_{\mathrm{TE}}), L_L = \frac{1}{\sqrt{2}}(L_{\mathrm{TM}} - iL_{\mathrm{TE}})\right)$ of right (L_R) and left circularly polarized light (L_L) propagating with a wave vector component $k_\perp \propto \sin\theta_L$ perpendicular to the z-axis. θ_L is the propagation angle between the light beam and the z axis, while L_{TM} and L_{TE} are the corresponding light modes with polarization in the $x-z$ plane (TM) and perpendicular to it (TE), respectively. Due to the dependence of the light propagation through the DBRs on polarization direction, the L_{TM} and L_{TE} eigenstates are, in general, non-degerate for $\theta_L \neq 0$ [6]. The energy of the bare photon states in a trap can be described by the following Hamiltonian in the basis $(L_{\mathrm{TM}}, L_{\mathrm{TE}})$ [7]:

$$\begin{aligned} H_{LT}^{(p_xp_y)}(k_{av}) &= \frac{\hbar^2 k_{av}^2}{2m_{ph}} + \frac{\Delta_{\mathrm{LT}}(k_{av})}{k_{av}^2}\left[\begin{array}{c}(p_xk_x)^2-(p_yk_y)^2\\ 2p_xk_xp_yk_y\end{array}\right]_{av} \\ &= \frac{2}{3}\left(p_x^2+p_y^2\right)E_{01} + \delta_{\mathrm{LT}}^{(p_xp_y)} \end{aligned} \tag{6}$$

$$k_{av}^2 = (p_xk_x)^2 + (p_yk_y)^2$$

$$\delta_{\mathrm{LT}}^{(p_xp_y)} = (\hbar\omega_{\mathrm{TM}} - \hbar\omega_{\mathrm{TE}}) \approx \Delta_{\mathrm{LT}}(k_{av},0)\left[\frac{p_x^2-p_y^2}{p_x^2+p_y^2} - 2\frac{p_xp_y}{p_x^2+p_y^2}\frac{\Delta a}{a}\right]. \tag{7}$$

where m_{ph} is the bare photon mass. In the second line of Eq. 6, the confinement energy shift of the bare photonic level is expressed in terms of the polariton interlevel splitting (in a manner analogous to the excitonic states in Eq. 5) assuming that the bare photonic mass in the MC is half the polariton mass m_p (i.e., $m_{ph} \approx m_p/2$).

The longitudinal-transverse splitting $\Delta_{\mathrm{LT}}(k_{av},0)$ depends on the photon wave vector as well as on the layer structure of the MC. When averaged over the wave function of a polariton mode (p_xp_y), the energy splitting between the L_{TM} (with energy $\hbar\omega_{\mathrm{TM}}$) and L_{TE} ($\hbar\omega_{\mathrm{TE}}$) states becomes equal to $\delta_{\mathrm{LT}}^{(p_xp_y)}$ given by Eq. 7. This approximation on the right-hand side applies for $\delta a/a \ll 1$.

The pseudo-spin splitting Δ_{LT} in Eq. 7 corresponds to the splitting between the bare photonic states for light propagation with an in-plane wave vector k_x. Figure 11 displays the average energy $(\hbar\omega_{\mathrm{TM}}(\vec{k}) + \hbar\omega_{\mathrm{TE}}(\vec{k}))/2$ as well as $\Delta_{\mathrm{LT}}(\vec{k}) = (\hbar\omega_{\mathrm{TM}}(\vec{k}) - \hbar\omega_{\mathrm{TE}}(\vec{k}))$ calculated for the $(p_xp_y) = (1,1)$ polariton mode with a wave vector $\vec{k} = (k_x, 0)$ in the sample structure used in this work. The calculations were carried out using a transfer matrix approach to determine the energy of the light states in the $x--z$ plane with polarization along y (TE-polarization) and in the incidence plane (TM-polarization) in an empty MC, i.e., without including excitonic contributions to the dielectric response. For small propagation angles, both the average energy and the pseudo-spin splitting increase quadratically with the wave vector. The dashed vertical line marks the wave vector component $k_x = \pi/a_x$ corresponding to the polariton GS of a 4×4 $\mu\mathrm{m}^2$ trap. From the splitting $\Delta_{\mathrm{LT}}(k) = 9.5$ μeV indicated by the dashed line, one obtains

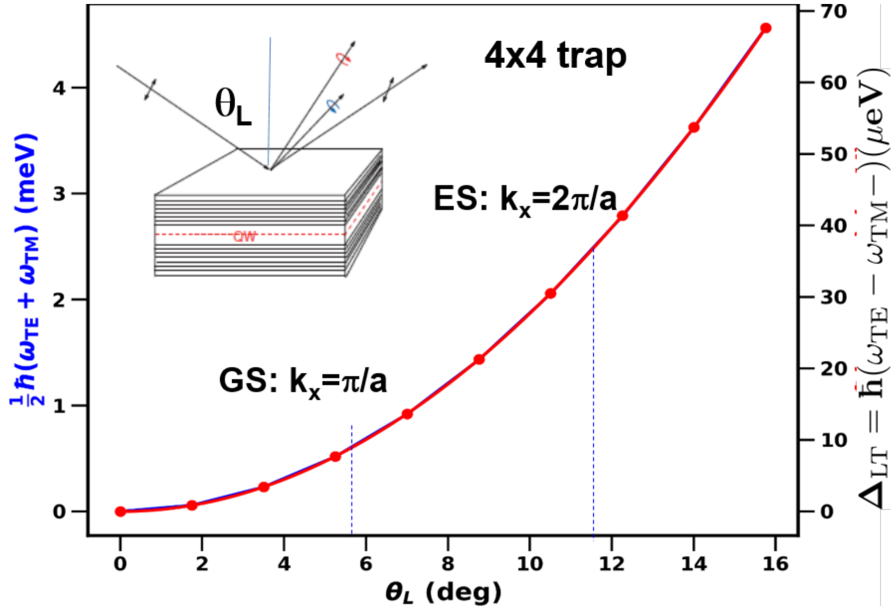

Fig. SM 11: **Energy and splitting calculations.** Calculated average energy ($(\hbar\omega_{\rm TE} + \hbar\omega_{\rm TM})/2$, left vertical scale) as well as the splitting $\delta_{\rm TE-TM} = \hbar(\omega_{\rm TE} - \omega_{\rm TM})$ between transverse electric (TE) and transverse magnetic (TM) modes of the optical microcavity of Supplementary Table 1 as a function of the angle of incidence θ_L (relative to the z axis) in the $x - z$ plane. The dashed vertical line marks θ_L corresponding to an in-plane wave vector $\vec{k} = (k_{\rm x}, k_{\rm y}) = (\pi/a, 0)$ for the $(p_x, p_y) = (1, 1)$ polariton mode yielding a splitting of 9.5 μeV, where $a = 4$ μm is the trap size.

from Eq. 7 energy splittings $\delta_{\rm LT}^{(1,1)} = 0.48$ μeV and $\delta_{\rm LT}^{(2,1)} = 3.6$ μeV for the GS and first excited state (ES) of a trap with $\Delta a/a = 10\%$. In both cases, the splitting is much smaller than the 3λ LA phonon quantum of $\hbar\omega_m = 28$ μeV.

B. Confined phonon modes

Similarly to the case of polaritons, we will assume the displacement field $\vec{u}(x, y, z)$ of the phonons to be confined within a box with lateral sizes corresponding to the trap size and dimension along z equal to the thickness of the cavity spacer $m_s\lambda_{\rm BAW}/2$, as illustrated in Fig. 10.

The confined phonon modes at the QW plane can be approximated by those of a rectangular box with dimensions $a_x \sim a_y$ along the x and y directions, respectively. If the lateral surfaces of the box at $x = \pm a_x/2$ and $y = \pm a_y/2$ are free to move, the displacement field $\vec{u}(x, y, z)$ for the three eigenmodes is $\left(\vec{u}^{TA'}_{(\rm m_x m_y)}, \vec{u}^{TA}_{(\rm m_x m_y)}, \vec{u}^{LA'}_{(\rm m_x m_y)}\right)$ with a wave vector $k_z = m_z\pi/\lambda_{\rm BAW}$ along $z||[001]$. The indices $(m_x m_y)$, $m_i = 1, 2, \ldots, i = x, y$ describe the number of the transverse lobes (i.e., perperdincular to z) of the acoustic mode. m_z is the index of the longitudinal mode being equal to 1 and 3 for the λ- and 3λ phonon modes, respectively. The displament field of these modes can be stated as the columns of the following matrix:

$$\vec{u}_{(\rm m_x m_y)}(x, y, z) = \left(\vec{u}^{TA'}_{(\rm m_x m_y)}, \vec{u}^{TA}_{(\rm m_x m_y)}, \vec{u}^{LA'}_{(\rm m_x m_y)}\right) \tag{8}$$

$$= u_{zz_0} \begin{pmatrix} \frac{r_T}{k_x m_x} & \frac{1}{k_x m_x} & -\frac{k_x m_x}{2k_z^2 r_T} \\ \frac{r_T}{k_y m_y} & -\frac{1}{k_y m_y} & -\frac{k_y m_y}{2k_z^2 r_T} \\ \frac{1}{k_z} & 0 & \frac{1}{k_z} \end{pmatrix} e^{i(k_x m_x x + k_y m_y y + k_z z)},$$

where

$$r_T = c_{12}/(c_{11} + c_{12}). \tag{9}$$

is the ratio between the uniaxial deformation perpendicular and parallel to the z axis. For GaAs, $r_T = -0.31$. The amplitude of the acoustic field in Eq. 8 is stated in terms of the amplitude $u_{zz,0}$ of the uniaxial strain along z, which, as previously mentioned, has a maximum close to the QW position.

The $\vec{u}^{TA}_{(\mathrm{m_x m_y})}(x,y,z)$ eigenmode in Eq. 8 is a pure transversal acoustic (TA) mode polarized in the x-y plane of the trap and angular frequency $f_{TA} \sim \frac{1}{2\pi}\sqrt{\frac{c_{44}}{\rho}}k_z$. The two other modes have mixed transversal and longitudinal acoustic (LA) character induced by the lateral confinement. The mixing is dictated by r_T as well as by the dimensions of the trap. For large traps (i.e., $a \gg \lambda_{\mathrm{BAW}}$), $\vec{u}^{TA'}_{(\mathrm{m_x m_y})}(x,y,z)$ can be well approximated by a TA wave with a small displacement component along z. Its angular frequency is slighly smaller than f_{TA}. $\vec{u}^{LA'}_{(\mathrm{m_x m_y})}(x,y,z)$ is essentially a LA mode with a small transverse component and angular frequency $f_{LA} \sim \frac{1}{2\pi}\sqrt{\frac{c_{11}}{\rho}}k_z$.

The frequency of the 3λ phonon modes for different $(m_x m_y)$ combinations are listed in the first two columns of Supplementary Table 5. These frequencies were determined using the elastic properties of GaAs. Since the spacer of the MC also includes (Al,Ga)As layers, which have higher acoustic velocities, the listed values sligthly underestimate the measured resonance frequencies. The corresponding frequencies for the λ phonons are three times as large. To a very good approximation (i.e., with deviations less than $\sim 5\%$), the relationship between the mode frequencies is $f_{TA'} \approx f_{TA} \approx 0.7 f_{\mathrm{LA}}$ for modes with $m_i \leq 2$ $(i = 1, 2)$. It is interesting to note that the frequencies $f_{TA'}$ and f_{TA} for the λ phonons are approximately twice as large as the frequency f_{LA} for the 3λ phonons. By solving the elastic equations for a 4×4 μtm^2 trap, we obtain the following frequency ratios:

$$f^{(\lambda)}_{TA'} \approx f^{(\lambda)}_{TA} = 2.07 f^{(3\lambda)}_{LA} = 2.07 f_{\mathrm{BAW}} \tag{10}$$

$$f^{(\lambda)}_{LA'} = 2.95 f^{(\lambda)}_{LA} = 2.95 f_{\mathrm{BAW}}, \tag{11}$$

where f_{BAW} is the frequency of the phonons generated by the BAWRs. The λ-phonon frequencies are indicated by thick arrows in Figs. 6, 7 and 8 of this supplement.

The vibrational eigenmodes of the trap are superpositions of the modes of Eq. 8 satisfying the boundary conditions at the trap borders. Here, we will assume that the uniaxial strain components vanish at the lateral trap borders. In this case, we obtain the following expression for the displacement field of mode $\vec{u}^{LA'}_{(\mathrm{m_x m_y})}(x,y,z)$, which is also the one that can be efficiently excited by the BAWRs (similar expression can be easily derived for the other modes from Eq. 8):

$$\vec{u}_{(\mathrm{m_x m_y})}(x,y,z) = \vec{u}^{LA'}_{(\mathrm{m_x m_y})}(x,y,z) = \begin{pmatrix} \frac{k_x m_x \cos(k_z z)\cos\left(\frac{1}{2}m_x(\pi-2k_x x)\right)\sin\left(\frac{1}{2}m_y(\pi-2k_y y)\right)}{2k_z^2 r_T} \\ \frac{k_y m_y \cos(k_z z)\sin\left(\frac{1}{2}m_x(\pi-2k_x x)\right)\cos\left(\frac{1}{2}m_y(\pi-2k_y y)\right)}{2k_z^2 r_T} \\ -\frac{\sin(k_z z)\sin\left(\frac{1}{2}m_x(\pi-2k_x x)\right)\sin\left(\frac{1}{2}m_y(\pi-2k_y y)\right)}{k_z} \end{pmatrix} u_{zz_0} \tag{12}$$

The changes in the lattice induced by the phonon field are proportional to the displacement gradient $\nabla\vec{u}_{(m_x m_y)}(x,y)$. The latter can be decomposed into a symmetric ($\varepsilon_{m_x m_y} = \frac{1}{2}\left[\nabla u_{(m_x m_y)} + (\nabla u_{(m_x m_y)})^T\right]$) and an anti-symmetric ($\vec{\omega}_{(m_x m_y)} = \frac{1}{2}\left[\nabla u_{(m_x m_y)} - (\nabla u_{(m_x m_y)})^T\right]$) contribution. The first corresponds to the strain field while the second yields a pure rotation of the lattice (i.e., without the lattice distortion). $\vec{\omega}_{(m_x m_y)}$ is much smaller than $\varepsilon_{m_x m_y}$ and will be neglected in the next sections.

The amplitude of the strain field $u_{zz,0}$ in Eq. 12 can be determined by calculating the energy stored in the strain field. This amplitude $u_{zz,0}$ can be stated in terms of the phonon frequency $\hbar\omega_{\mathrm{mec}}$, and number n_{mec}, and the zero-point motion $u^{ZPM}_{zz,0}$ as:

$$u_{zz,0} = \sqrt{n_{\mathrm{mec}}}\,u^{ZPM}_{zz,0}, \quad u^{ZPM}_{zz,0} = \frac{64\sqrt{f_{\mathrm{BAW}}}}{a_x a_y}\sqrt{\frac{1}{128\rho v^3_{LA} + \frac{8\rho {v_{LA}}^7}{(a_x a_y)^4} f^4_{\mathrm{BAW}} {r_T}^2}}. \tag{13}$$

The second term in the denominator within the square root is the correction introduced by the reduced trap size.

Figures 12(a) and 12(b) display the amplitude of the strain (u^{ZPM}_{zz}) and displacement field component (u^{ZPM}_{z}), respectively, calculated using Eq. 13 for a single phonon confined in a 4×4 μm^2 trap with different thicknesses $\lambda_{\mathrm{BAW}}/2$ (and, thus different frequencies f_{BAW}). For short phonon wavelengths (i.e., frequencies above ~ 5 GHz) all modes indexed by $(m_x m_y)$ have approximately the same frequency dependence with the strain (displacement) field proportional to $f^{1/2}_{\mathrm{BAW}}$ $(1/f^{1/2}_{\mathrm{BAW}})$. For a 7 GHz phonons investigated in this work, the strain amplitudes $u^{\mathrm{ZPM}}_{zz} \sim 10^{-8}$ are expected to induce energy shifts of the excitonic electron-heavy hole transition of approximately $a_h u^{\mathrm{ZPM}}_{zz} \approx$ 0.10 μeV$= 2\pi\hbar$ 25 MHz, where a_h is the hydrostatic exciton deformation potential (cf. Supplementary Table 3).

Supplementary Table 3: Parameters used in the calculations.

Parameter	Value	Unit	Remarks
Material properties			unless specified, for GaAs
a_h	-9	eV	hydrostatic deformation potential [8]
b	-2	eV	uniaxial deformation potential [8]
d	-6	eV	shear deformation potential [8]
$c_{11}^{(c)}$	1.183×10^{11}	N/m^2	elastic constant [9]
$c_{12}^{(c)}$	0.532×10^{11}	N/m^2	elastic constant [9]
$c_{44}^{(c)}$	0.595×10^{11}	N/m^2	elastic constant [9]
ρ	5316.5	kg/m^3	density [9]
ϵ_{rel}	12	-	static dielectric constant
e_{14}	-0.16	C/m^2	piezoelectric strain coefficient [9]
Trap properties			
$a_{\rm trap}$	4	μm	trap size
$\Delta a_{\rm trap}$	-	μm	trap anisotropy (cf. Fig. 10)
Polariton properties			
$\delta_{\rm CX}$	0	meV	Cavity-exciton detuning
E_{01}	1	meV	splitting between the polariton GS and first ES
$\Delta_{\rm XY}$	100	μeV	Trap anisotropic energy (real part)
Δ_R	0.0	eV	Trap anisotropy energy (imag. part)
$\Delta_{\rm LT}$	9.5	μeV	LT splitting for the ground polariton $(p_x p_y) = (1,1)$ state
$\delta_{\rm LT}^{(1,1)}$	1.5	μeV	integrated LT splitting for the $(p_x p_y) = (1,1)$ state, cf. Eq. (7)
$\Delta_{\rm hh-lh}$	6	meV	QW lh-hh energy splitting
$\Omega_{\rm R}$	3	meV	polariton Rabi coupling for e-hh states
$\Gamma_{\rm pol}$	4.8	μeV	Polariton BEC decoherence rate
$N_{\rm pol}$	10^5		Polariton BEC population at the threshold
Phonon properties			
$f_{\rm mec}$	7	GHz	Frequency of piezoelectrically generated phonons
$\Gamma_{\rm mec}$	5.5×10^{-3}	μeV	Phonon decoherence rate

C. Phonon-polariton coupling

1. *Coupling mechanisms*

The phonon field acts on the polariton states in the three different ways illustrated schematically in Fig. 13. The solid and dashed lines for a polariton with orbital index $p_x p_y$ indicate the states with up ($\uparrow$) and down ($\downarrow$) pseudo-spin indices, respectively:

- The phonons can modulate the energy of the individual polariton states with the coupling strength $\delta E_{hh,(m_x m_y)}^{(p_x p_y)}$: if the energy modulation frequency exceeds the polariton linewidth, this modulation leads to the formation of emission sidebands.
- The phonons can mix the polariton pseudo-spin states $\Psi_{(p_x p_y)}^{(s)}(x,y)$ with the same orbital indices. This *intra-mode coupling* process is quantified by the coupling energy $g_{\uparrow\downarrow(m_x m_y)}^{(p_x p_y)}$).
- Alternatively, they can induce an *inter-mode coupling* between polaritons with different orbital indices. The coupling strengths for inter-mode spin-conserving and spin-flipping processes are denoted by $g_{\uparrow\uparrow(m_x m_y)}^{(p_x p_y)\leftrightarrow(p'_x p'_y)} = g_{\downarrow\downarrow(m_x m_y)}^{(p_x p_y)\leftrightarrow(p'_x p'_y)}$ and $g_{\uparrow\downarrow(m_x m_y)}^{(p_x p_y)\leftrightarrow(p'_x p'_y)}$ factors, respectively.

Details of the coupling mechanisms will be addressed in the next sections.

2. *Averaged strain field for polariton coupling*

We will restrict ourselves to phonon-polariton coupling relying on the deformation potential mechanism, which has been shown to dominate the coupling in (Al,Ga)As MCs [1]. In this framework, the effects of the acoustic field on the

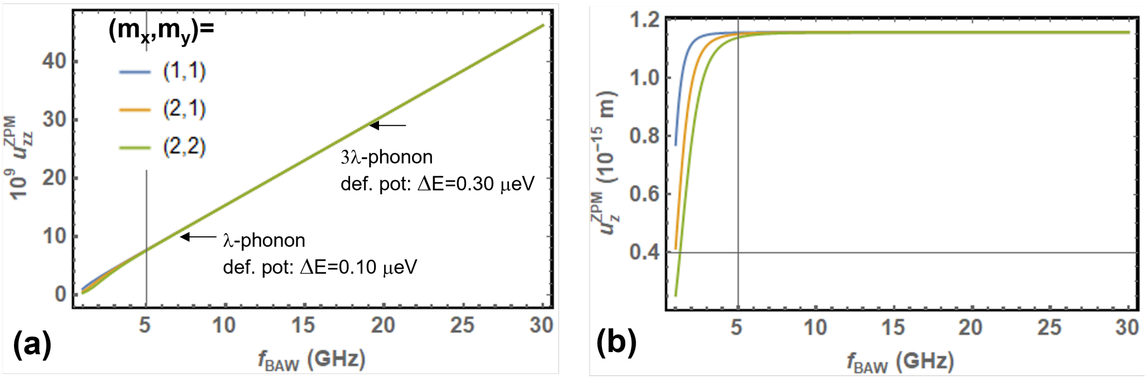

Fig. SM 12: **Strain calculations.** Calculated amplitude of the strain field component (a) u_{zz}^{ZPM} and of (b) the z-oriented displacement u_z^{ZPM} corresponding to the zero-point fluctuation of a phonon with indices $m_x m_y$ in a $4 \times 4\ \mu\mathrm{m}^2$ trap with different thicknesses $\lambda_{\mathrm{BAW}}/2$, which yield the phonon frequencies displayed by the horizontal axis.

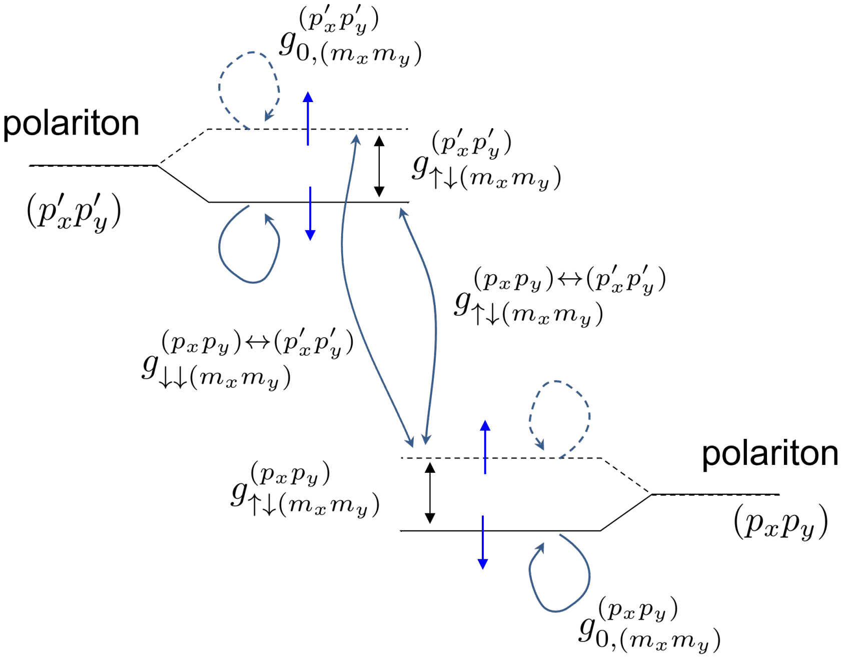

Fig. SM 13: **Polariton energy and phonon coupling diagram sketch.** Coupling between the polariton levels $(p_x p_y)$ and (p'_x, p'_y) mediated by a $(m_x m_y)$ phonon. Intra-level coupling induces the energy modulation of the pseudo-spin levels (quantified by the coupling strength $g_{0,(m_x m_y)}^{(p_x p_y)}$ and $g_{0,(m_x m_y)}^{(p'_x p'_y)}$) as well as coupling between the pseudo-spin states ($g_{\uparrow\downarrow(m_x m_y)}^{(p_x p_y)}$ and $g_{\uparrow\downarrow(m_x m_y)}^{(p'_x p'_y)}$). The inter-level coupling is mediated by the spin-conserving and non-conserving factors $g_{\uparrow\uparrow(m_x m_y)}^{(p_x p_y)\leftrightarrow(p'_x p'_y)} = g_{\downarrow\downarrow(m_x m_y)}^{(p_x p_y)\leftrightarrow(p'_x p'_y)}$ and $g_{\uparrow\downarrow(m_x m_y)}^{(p_x p_y)\leftrightarrow(p'_x p'_y)}$, respectively.

coupling of polariton mode $\Psi_{(p_x p_y)}^{(s)*}$ are mediated by its excitonic component with (orthonormalized) wave function $\chi_{(p_x p_y)}^{(s)*}$, which we will assume to have the same spatial dependence as the polariton wave function $\Psi_{(p_x p_y)}^{(s)*}$ in Eq. 2. The coupling elements can be stated as:

$$\langle\chi^{(s')*}_{(p'_x,p'_y)}|\nabla\vec{u}_{(m_x m_y)}(x,y)|\chi^{(s)}_{(p_x p_y)}\rangle = \varepsilon^{(p'_x,p'_y)\leftrightarrow(p_x p_y)}_{(m_x m_y)} + \vec{\omega}^{(p'_x,p'_y)\leftrightarrow(p_x p_y)}_{(m_x m_y)} \tag{14}$$

$$\varepsilon^{(p'_x,p'_y,s')\leftrightarrow(p_x p_y,s)}_{(m_x m_y)} = \int \varepsilon_{(m_x m_y)}\chi^{(s')*}_{(p'_x,p'_y)}(x,y)\chi^{(s)}_{(p_x p_y)}(x,y)dxdy \tag{15}$$

$$\vec{\omega}^{(p'_x,p'_y,s')\leftrightarrow(p_x p_y,s)}_{(m_x m_y)} = \int \vec{\omega}_{(m_x m_y)}\chi^{(s')*}_{(p'_x,p'_y)}(x,y)\chi^{(s)}_{(p_x p_y)}(x,y)dxdy. \tag{16}$$

From this point on, we will implicitly assume that the optoelectronic coupling is mediated by the excitonic component and no longer distinguish between $\chi^{(s)*}_{(p_x p_y)}$ and $\Psi^{(s)*}_{(p_x p_y)}$. It can be shown that the right-hand side of Eq. 15, which corresponds to the antisymmetric part related to rotation, vanishes when averaged over the excitonic wavefunction of the quasi-LA mode of Eq. 12.

We will be first interested in the effects of the strain from the $\vec{u}^{LA'}_{(\mathrm{m_x m_y})}$ confined phonons on the lower lying polariton levels corresponding to the pseudo-spin split ground state (GS) $(p_x p_y) = (1,1)$ and first excited states (ES) with $(p_x p_y) = (2,1)$, $(1,2)$, or $(2,2)$. These modes are expected to have the longest coherence time and smallest energy separations, which can be comparable to the phonon quantum. The relevant phonons are those with the lowest energy, which are well confined within the cavity [i.e., the $(m_x m_y) = (1,1)$ GS mode and the first ES modes with $(m_x m_y) = (2,1)$ or $(1,2)$].

Supplementary Table 4: Integrated effective strain $\varepsilon^{(p'_x,p'_y,s')\leftrightarrow(p_x p_y,s)}_{(m_x m_y)} = \left(e^c_{xx}, e^c_{yy}, e^c_{zz}, e^c_{yz}, e^c_{xz}, e^c_{xy}\right)$ in units of $\left(\frac{u_{zz}}{\pi^2}\right)$ associated with the phonon mode $\vec{u}^{LA'}_{(\mathrm{m_x m_y})}$ with $(m_x = 1, m_y = 1)$. The numerical values were calculated for a 4×4 $\mu\mathrm{m}^2$ trap with a shape anisotropy $\Delta a/a = 0.1$. Entries marked as "tr" in the matrix are equal to their transpose.

$(p_x p_y)$	$(p'_x p'_y) = (1,1)$	$(p'_x p'_y) = (1,2)$	$(p'_x p'_y) = (2,1)$	$(p'_x p'_y) = (2,2)$
11	$\begin{pmatrix} -\frac{128 r_T}{27} & 0 & \frac{64k_\perp^2}{27k_z^2 r_T} \\ -\frac{128 r_T}{27} & 0 & \frac{64k_\perp^2}{27k_z^2 r_T} \\ -\frac{128}{27} & 0 & -\frac{128}{27} \\ 0 & 0 & 0 \\ 0 & 0 & 0 \\ 0 & \frac{256}{27} & \frac{128k_\perp^2}{27k_z^2 r_T}\frac{\Delta a}{a} \end{pmatrix}$	tr	tr	tr
12	0	$\begin{pmatrix} -\frac{512 r_T}{135} & 0 & \frac{256k_\perp^2}{135k_z^2 r_T} \\ -\frac{512 r_T}{135} & 0 & \frac{256k_\perp^2}{135k_z^2 r_T} \\ -\frac{512}{135} & 0 & -\frac{512}{135} \\ 0 & 0 & 0 \\ 0 & 0 & 0 \\ 0 & \frac{1024}{135} & \frac{512k_\perp^2}{135k_z^2 r_T}\frac{\Delta a}{a} \end{pmatrix}$	tr	tr
21	0	$\begin{pmatrix} \frac{\pi^2 r_T}{6} & \frac{\pi^2}{6}\frac{\Delta a}{a} & -\frac{\pi^2 k_\perp^2}{12k_z^2 r_T} \\ -\frac{1}{6}\pi^2 r_T & -\frac{\pi^2}{6}\frac{\Delta a}{a} & \frac{\pi^2 k_\perp^2}{12k_z^2 r_T} \\ 0 & 0 & 0 \\ 0 & 0 & 0 \\ 0 & 0 & 0 \\ 0 & 0 & 0 \end{pmatrix}$	$\begin{pmatrix} -\frac{512 r_T}{135} & 0 & \frac{256k_\perp^2}{135k_z^2 r_T} \\ -\frac{512 r_T}{135} & 0 & \frac{256k_\perp^2}{135k_z^2 r_T} \\ -\frac{512}{135} & 0 & -\frac{512}{135} \\ 0 & 0 & 0 \\ 0 & 0 & 0 \\ 0 & \frac{1024}{135} & \frac{512k_\perp^2}{135k_z^2 r_T}\frac{\Delta a}{a} \end{pmatrix}$	0
22	$\begin{pmatrix} \frac{\pi^2 r_T}{6} & \frac{\pi^2}{6}\frac{\Delta a}{a} & -\frac{\pi^2 k_\perp^2}{12k_z^2 r_T} \\ -\frac{1}{6}\pi^2 r_T & -\frac{\pi^2}{6}\frac{\Delta a}{a} & \frac{\pi^2 k_\perp^2}{12k_z^2 r_T} \\ 0 & 0 & 0 \\ 0 & 0 & 0 \\ 0 & 0 & 0 \\ 0 & 0 & 0 \end{pmatrix}$	0	0	$\begin{pmatrix} -\frac{2048 r_T}{675} & 0 & \frac{1024k_\perp^2}{675k_z^2 r_T} \\ -\frac{2048 r_T}{675} & 0 & \frac{1024k_\perp^2}{675k_z^2 r_T} \\ -\frac{2048}{675} & 0 & -\frac{2048}{675} \\ 0 & 0 & 0 \\ 0 & 0 & 0 \\ 0 & \frac{4096}{675} & \frac{2048k_\perp^2}{675k_z^2 r_T}\frac{\Delta a}{a} \end{pmatrix}$

(17)

Expressions can be derived from Eqs. 12 and 15 for the strain field components $\varepsilon_{(m_x m_y)}^{(p'_x,p'_y,s')\leftrightarrow(p_x p_y,s)}$ induced by a $(m_x m_y)$ phonon and averaged over the polariton modes (p'_x, p'_y) and $(p_x p_y)$. As an example, Supplementary Table 4 summarizes the average strain fields associated with GS phonon mode $(m_x m_y) = (1,1)$. The strain tenson is listed in engineering notation with $\varepsilon^c_{(m_x m_y)} = \left(e^c_{xx}, e^c_{yy}, e^c_{zz}, e^c_{yz}, e^c_{xz}, e^c_{xy}\right)$ in the cartesian reference frame (superscript ^{c}). We use the representation in the cartesian frame to obtain a more familiar representation of the Pikus and Bir Hamiltonian in Sec. V C 3.

The following points related to the $\varepsilon_{(m_x m_y)}^{(p'_x,p'_y,s')\leftrightarrow(p_x p_y,s)}$ are worth mentioning:

- A phonon mode with an uneven (even) index m_i induces a strain field with even (uneven) orbital parity along the i axis. In a perfectly square trap, this phonon can thus only efficiently couple polariton modes with the same (different) parity with respect to the i axis. The vanishing $\varepsilon_{(m_x m_y)}^{(p'_x,p'_y,s')\leftrightarrow(p_x p_y,s)}$ terms in Supplementary Table 4 result from combinations of indices, for which this condition is not satisfied.

- As a consequence, phonons with uneven indices can efficiently modulate the energy of the individual states by inducing a strong coupling $g_{0,(m_x m_y)}^{(p_x p_y)}$ (cf. Fig. 13) . This modulation amplitude, which is normally dominated by the e^c_{zz} component, has an important role in the formation of sidebands.

- Phonons with even indices, in contrast, lead to low $g_{0,(m_x m_y)}^{(p_x p_y)}$ couplings. These phonons can, however, induce couplings between levels with different orbital indices with a strength $g_{\uparrow\uparrow(\uparrow\downarrow),m_x m_y}^{p_x p_y \leftrightarrow p'_x p'_y}$ mainly determined by the e^c_{zz} and e^c_{xz} components for spin-conserving and spin-flipping coupling (see below). As an example, a $(m_x m_y) = (2,2)$ phonon can couple both ES modes (such as $(p_x p_y) = (2,1)$ and $(1,2)$) as well as the GS and ES polaritons $(p_x p_y) = (1,1)$ and $(2,2)$ (see Supplementary Table 5). The latter creates a channel for polariton relaxation from the ES to the GS with phonon emission. The coupling is not resonant since the splitting between the polariton states is normally much larger than the phonon energy.

- The uniaxial components e^c_{xx}, e^c_{yy}, e^c_{zz} do not reduce the symmetry of traps below the tetragonal one and can, therefore, not couple states with different pseudo-spins. This coupling requires shear components. In a perfectly square trap (i.e., with $\Delta a = 0$), the only non-vanishing shear component is the e^c_{xy} one induced by the TA mode (this result also applies for other phonons). The TA phonons are, therefore, the only ones able to generate a $g_{\uparrow\downarrow,m_x m_y}^{p_x p_y \leftrightarrow p'_x p'_y}$ coupling for the efficient interaction of states with different pseudo-spin. A trap distortion can introduce a small component $e^c_{xy} \propto \Delta a/a$ for the LA' mode.

3. *Strain effects on excitonic states*

The electron states forming the excitons have a simple s-type electronic wavefunction, which is only sensitive to the hydrostatic component of the strain field. The valence band states, in contrast, are superpositions of p-like $\langle X\rangle$, $\langle Y\rangle$, and $\langle Z\rangle$ orbitals, which become mixed by the strain. The relevant excitonic levels are those associated with the lowest lying electron-heavy hole (hh) and electron-light hole (lh) transitions in the QWs formed by linear superpositions of the s and p-orbitals. We describe these states in a basis of valence band states angular and spin momenta $\langle m_j, s\rangle$ with $j = 3/2$ and $m = 1/2$ given by $(X_{hh\Uparrow}, X_{lh\Downarrow}, X_{lh\Uparrow}, X_{hh\Downarrow})$, which mix with the conduction band electrons to form the excitons.

The effects of the strain on the excitonic states are described by the well-known Pikus and Bir (PB) hamiltonian[10], which in the basis $(X_{hh\Uparrow}, X_{lh\Downarrow}, X_{lh\Uparrow}, X_{hh\Downarrow})$ becomes:

$$\mathbf{H}_{\rm PB} = \begin{pmatrix} \delta E_{\rm hh} & \frac{d}{2}(e^c_{xy} - i d e^c_{xz}) & \frac{1}{2} d e^c_{yz} & 0 \\ \frac{d}{2}(e^c_{xy} + i d e^c_{xz}) & \delta E_{\rm lh} & 0 & \frac{1}{2} d e^c_{yz} \\ \frac{1}{2} d e^c_{yz} & 0 & \delta E_{\rm lh} & -\frac{d}{2}(e^c_{xy} - i d e^c_{xz}) \\ 0 & \frac{1}{2} d e^c_{yz} & -\frac{d}{2}(e^c_{xy} + i d e^c_{xz}) & \delta E_{\rm hh} \end{pmatrix} \begin{pmatrix} X_{hh\Uparrow} \\ X_{lh\Downarrow} \\ X_{lh\Uparrow} \\ X_{hh\Downarrow} \end{pmatrix}, \quad (18)$$

where

$$\delta E^{(p_x p_y)}_{\rm hh,(m_x m_y)} = \delta E_{\rm hh} = (2e^c_{xx} + e^c_{zz}) a_h + (e^c_{zz} - e^c_{xx}) b \quad (19)$$

$$\delta E^{(p_x p_y)}_{\rm lh,(m_x m_y)} = \delta E_{\rm lh} = (2e^c_{xx} + e^c_{zz}) a_h - (e^c_{zz} - e^c_{xx}) b + \Delta_{\rm hh-lh}. \quad (20)$$

In the previous expressions, $\Delta_{\mathrm{hh-lh}}$ is the energy splitting between the lh and hh states and the strain components e^c_{ij} are obtained from $\varepsilon^{(p_xp_y)}_{(m_xm_y)} = (e^c_{xx}, e^c_{xx}, e^c_{zz}, e^c_{yz}, e^c_{xz}, e^c_{xy})^T$ in Eq. 15 and displayed for the $(m_xm_y) = (1, 1)$ phonon mode in Supplementary Table 5. a_h, b, and d are, respectively, the hydrostatic, uniaxial, and shear deformation potentials for the GaAs QWs listed in Supplementary Table 3. In the right-most side of the equation, N_{mec} is the number of phonons and $g^{(p_xp_y)}_{0,(\mathrm{m_xm_y})}$ the energy modulation strength by a single phonon. To simplify the notation, we will eliminate the phonon and polariton indices of $\delta E^{(p_xp_y)}_{\mathrm{hh},(\mathrm{m_xm_y})}$ and $\delta E^{(p_xp_y)}_{\mathrm{lh},(\mathrm{m_xm_y})}$ whenever these can be inferred from the context. Note that the uniaxial strain components along the diagonal modulate the energy of the hh and lh states, while the shear ones introduce a reduction in symmetry that couples these states.

By including the exciton-photon coupling with an energy Ω_R (and $\Omega_R/3$ for the lh states due to the reduced oscillator strength), the polariton Hamiltonian H_{Pol} in the $(X_{hh\Uparrow}, X_{lh\Downarrow}, X_{lh\Uparrow}, X_{hh\Downarrow}, L_L, L_R)$ basis becomes:

$$\mathbf{H}_{\mathrm{Pol}}\Psi_{Pol} = \begin{pmatrix} \delta E_{\mathrm{hh}} & \frac{1}{2}d(e^c_{xy} - ie^c_{xz}) & \frac{1}{2}de^c_{yz} & 0 & 0 & -\Omega_{\mathrm{R}} \\ \frac{1}{2}d(e^c_{xy} + ie^c_{xz}) & \delta E_{\mathrm{lh}} & 0 & \frac{1}{2}de^c_{yz} & 0 & -\frac{\Omega_{\mathrm{R}}}{3} \\ \frac{1}{2}de^c_{yz} & 0 & \delta E_{\mathrm{lh}} & -\frac{1}{2}d(e^c_{xy} - ie^c_{xz}) & \frac{\Omega_{\mathrm{R}}}{3} & 0 \\ 0 & \frac{1}{2}de^c_{yz} & -\frac{1}{2}d(e^c_{xy} + ie^c_{xz}) & \delta E_{\mathrm{hh}} & \Omega_{\mathrm{R}} & 0 \\ 0 & 0 & \frac{\Omega_{\mathrm{R}}}{3} & \Omega_{\mathrm{R}} & \delta_{\mathrm{CX}} & \frac{i\delta_{\mathrm{LT}}}{2} \\ -\Omega_{\mathrm{R}} & -\frac{\Omega_{\mathrm{R}}}{3} & 0 & 0 & -\frac{i\delta_{\mathrm{LT}}}{2} & \delta_{\mathrm{CX}} \end{pmatrix} \begin{pmatrix} X_{hh\Uparrow} \\ X_{lh\Downarrow} \\ X_{lh\Uparrow} \\ X_{hh\Downarrow} \\ L_L \\ L_R \end{pmatrix}. \quad (21)$$

The upper right 4×4 matrix block corresponds to the exciton Hamiltonian of Eq. 18. In the lower left 2×2 block, the diagonal term δ_{CX} is the detuning between the bare photon and exciton states while the off-diagonal terms describe the mixing of the photonic states induced by the TE-TM coupling discussed in the previous section.

We are interested in the lowest energy eigenstates of Eq. 21, as well as on their mixing by the strain field. In order to obtain an analytical expression for these modes, we proceed with a series of simplification steps. We first note that the lh states are blue-shifted relative to the hh states by an energy $\sim \Delta_{\mathrm{hh-lh}}$ much larger than the phonon energy. To address the polariton-phonon coupling, we can then elliminate the lh states from the basis by using perturbartion theory to include their effects on the coupling between the hh and light states. In this way, the dimension of the basis reduces from 6 to 4. Furthermore, we further simplify the expressions by taking into account that the strain components e_{xz} and e_{yz} vanish for the considered polariton modes (cf. Supplementary Table 4). The simplified interaction matrix in the reduced basis $(X_{hh\Uparrow}, X_{hh\Downarrow}, L_L, L_R)$ after these transformations reads:

$$\mathbf{H}_{\mathrm{c}}\Psi_c = \begin{pmatrix} \delta E_{\mathrm{hh}} & 0 & 0 & -\Omega_{\mathrm{R}} + \frac{\Omega_{\mathrm{R}} de^c_{xy}}{\Delta_{\mathrm{hh-lh}}} \\ 0 & \delta E_{\mathrm{PB,hl}} & \Omega_{\mathrm{R}} + \frac{\Omega_{\mathrm{R}} de^c_{xy}}{6\Delta_{\mathrm{hh-lh}}} & 0 \\ 0 & \Omega_{\mathrm{R}} + \frac{\Omega_{\mathrm{R}} de^c_{xy}}{6\Delta_{\mathrm{hh-lh}}} & \delta_{\mathrm{CX}} & \frac{i}{2}\delta^{(p_xp_y)}_{\mathrm{LT}} \\ -\Omega_{\mathrm{R}} + \frac{\Omega_{\mathrm{R}} de^c_{xy}}{\Delta_{\mathrm{hh-lh}}} & 0 & -\frac{i}{2}\delta^{(p_xp_y)}_{\mathrm{LT}} & \delta_{\mathrm{CX}} \end{pmatrix} \begin{pmatrix} X_{hh\Uparrow} \\ X_{hh\Downarrow} \\ L_L \\ L_R \end{pmatrix}. \quad (22)$$

Equation 22 can be analytically diagonalized in the absence of the strain field (i.e., for $\varepsilon_{ij} = 0$) and used to determined the unitary transformation matrix in the polariton basis. Furthermore, phonon-mediated interactions between the upper (UP) and lower (LP) polariton states can be neglected since the energy difference between these two states is much larger than the phonon energy. In this way, we arrive to the following interaction Hamiltonian for the phonon-induced coupling between the LP pseudo-spin states $\left(\Psi^{(p_xp_y)}_{\mathrm{LP}\Uparrow}, \Psi_{\mathrm{LP}\Downarrow}\right)$:

$$\begin{aligned} \mathbf{H}^{(\mathbf{p_xp_y})}_{\mathrm{LP},(\mathrm{m_xm_y})} &= \mathbf{H}^{(\mathbf{p_xp_y})^{\Uparrow} \leftrightarrow (\mathbf{p_xp_y})^{\Downarrow}}_{\mathrm{LP},(\mathrm{m_xm_y})} \\ &= \left[-\Omega_R + \frac{1}{2}\delta_{\mathrm{CX}} + \frac{1}{3}(p_x^2 + p_y^2)E_{01}\right] \begin{pmatrix} 1 & 0 \\ 0 & 1 \end{pmatrix} \\ &+ \begin{pmatrix} \frac{1}{4}\delta^{(p_xp_y)}_{\mathrm{LT}} + \frac{2\delta_{\mathrm{CX}} - \delta^{(p_xp_y)}_{\mathrm{LT}} + 4\Omega_R}{8\Omega_R}\delta E_{\mathrm{hh}} & \frac{\Omega_R}{6\Delta_{\mathrm{hh-lh}}} de^c_{\mathrm{xy}} \\ \frac{\Omega_R}{6\Delta_{\mathrm{hh-lh}}} de^c_{\mathrm{xy}} & -\frac{1}{4}\delta^{(p_xp_y)}_{\mathrm{LT}} + \frac{2\delta_{\mathrm{CX}} + \delta^{(p_xp_y)}_{\mathrm{LT}} + 4\Omega_R}{8\Omega_R}\delta E_{\mathrm{hh}} \end{pmatrix}. \end{aligned} \quad (23)$$

From the diagonal elements of the previous equation, one obtains the following approximation for the time-averaged splitting between the lowest energy eigenvalues ($E_\Uparrow$ and $E_\Downarrow$):

$$\Delta E^{(p_xp_y)} = E_{\Uparrow} - E_{\Downarrow} \approx \delta_{\rm LT}^{(p_xp_y)} \left(\frac{1}{2} - \frac{1}{4}\frac{\delta_{\rm CX}}{\Omega_R},\right) \tag{24}$$

which is valid for $\delta_{\rm CX} \ll \Omega_R$.

4. Intra-level coupling

The on-site energy $g_{0,\uparrow\uparrow(m_xm_y)}^{(p_xp_y)}$ as well as the coupling energy between the pseudo-spin states $g_{0,\uparrow\downarrow(m_xm_y)}^{(p_xp_y)}$ induced by a single phonon can be determined directly from the diagonal and non-diagonal elements of Eq. 23, respectively:

$$g_{0,\uparrow\uparrow(m_xm_y)}^{(p_xp_y)} = \left(\frac{1}{2} + \frac{\delta_{\rm CX}}{2\Omega_R}\right)\left[(2e_{xx}^{\rm c,ZPM} + e_{zz}^{\rm c,ZPM})a_h + (e_{zz}^{\rm c,ZPM} - e_{xx}^{\rm c,ZPM})b\right] \tag{25}$$

$$g_{0,\uparrow\downarrow(m_xm_y)}^{(p_xp_y)} = \frac{\Omega_R}{6\Delta_{\rm hh-lh}} d e_{xy}^{\rm c,ZPM}. \tag{26}$$

In these expressions, the superscripts ZPMR specify that the strain components are evaluated for a single phonon and we assumed that $|\delta_{\rm LT}^{(p_xp_y)}| \ll \Omega_R$. They justify our previous assertion that the uniaxial strain components defining the diagonal element δE_{hh} modulate the energy of the polariton states while the shear component couple their pseudo-spin counterparts $\Psi_{\rm LP\Uparrow}^{(p_xp_y)}$ and $\Psi_{\rm LP\Downarrow}^{(p_xp_y)}$ states. We note that $e_{xy}^{\rm c,ZPM}$ and, thus, the pseudo-spin coupling $g_{0,\uparrow\downarrow(m_xm_y)}^{(p_xp_y)}$, vanishes for the TA' and LA' modes in square traps (i.e., with $\Delta a/a = 0$), as well as for uneven phonons. For the TA mode, in contrast, $g_{0,\uparrow\downarrow(m_xm_y)}^{(p_xp_y)}$ is large and not sensitive to the trap asymmetry.

Equations 25 and 26 apply for the first-order coupling between pseudo-spin states. In the presence of stimulated phonons (e.g., induced by a BAWR), one can also envisage second-order coupling effects between pseudo-spin levels split by twice the phonon energy Ω_M with an effective coupling strength:

$$G_{2,\uparrow\downarrow(m_xm_y)}^{(p_xp_y)} = \frac{g_{0,\uparrow\downarrow(m_xm_y)}^{(p_xp_y)} g_{0,(m_xm_y)}^{(p_xp_y)}}{\hbar\Omega_M}. \tag{27}$$

Here, the polariton states emits (or absorbs) two phonons, the first in a spin-conserving and the second in a spin-flipping transition. Such a second-order process becomes relevant when $G_{2,\uparrow\downarrow(m_xm_y)}^{(p_xp_y)} \ll g_{0,\uparrow\downarrow(m_xm_y)}^{(p_xp_y)}$.

5. Inter-level coupling

The framework of the previous section can be readily extended to address the inter-level coupling mechanism between polariton levels with different orbital indices (p_xp_y) and (p'_x, p'_y) mediated by a (m_xm_y) phonon. The interaction between these states is described by the following Hamiltonian:

$$\mathbf{H}_{\rm LP,(m_xm_y)}^{(\mathbf{p_xp_y})\leftrightarrow(\mathbf{p'_x},\mathbf{p'_y})} = \begin{pmatrix} \mathbf{H}_{\rm LP,(m_xm_y)}^{(\mathbf{p_xp_y})^{\Uparrow}\leftrightarrow(\mathbf{p_xp_y})^{\Downarrow}} & \mathbf{H}_{\rm LP,(m_xm_y)}^{(\mathbf{p_xp_y})^{\Uparrow}\leftrightarrow(\mathbf{p'_xp'_y})^{\Downarrow}}\Big|_{\mathbf{mec}} \\ \mathbf{H}_{\rm LP,(m_xm_y)}^{(\mathbf{p'_xp'_y})^{\Uparrow}\leftrightarrow(\mathbf{p_xp_y})^{\Downarrow}}\Big|_{\mathbf{mec}} & \mathbf{H}_{\rm LP,(m_xm_y)}^{(\mathbf{p'_xp'_y})^{\Uparrow}\leftrightarrow(\mathbf{p'_xp'_y})^{\Downarrow}} \end{pmatrix}. \tag{28}$$

The 2×2 diagonal blocks of Eq. 28 are defined in Eq. 23. The 2×2 off-diagonal ones (i.e., $\mathbf{H}_{\rm LP,(m_xm_y)}^{(\mathbf{p_xp_y})\leftrightarrow(\mathbf{p'_x},\mathbf{p'_y})}\Big|_{\mathbf{mec}}$) are also obtained from Eq. 23 by neglecting the strain-independent terms. This equation defines the following two single-phonon coupling strengths for spin-conserving and a spin-flipping polariton transitions between the (p_xp_y) and (p'_x, p'_y) polariton states:

$$N_{mec} g_{0,\uparrow\uparrow,m_xm_y}^{p_xp_y\leftrightarrow p'_xp'_y} = \mathbf{H}_{\rm LP,(m_xm_y)}^{(\mathbf{p_xp_y})^{\Uparrow}\leftrightarrow(\mathbf{p_xp_y})^{\Downarrow}}|_{1,3} + \mathbf{H}_{\rm LP,(m_xm_y)}^{(\mathbf{p_xp_y})^{\Uparrow}\leftrightarrow(\mathbf{p_xp_y})^{\Downarrow}}|_{2,4} \tag{29}$$

$$N_{mec} g_{0,\uparrow\downarrow,m_xm_y}^{p_xp_y\leftrightarrow p'_xp'_y} = \mathbf{H}_{\rm LP,(m_xm_y)}^{(\mathbf{p_xp_y})^{\Uparrow}\leftrightarrow(\mathbf{p_xp_y})^{\Downarrow}}|_{1,4} + \mathbf{H}_{\rm LP,(m_xm_y)}^{(\mathbf{p_xp_y})^{\Uparrow}\leftrightarrow(\mathbf{p_xp_y})^{\Downarrow}}|_{2,3} \tag{30}$$

Supplementary Table 5: Coupling for polariton modes $(p_x p_y)$ and (p'_x, p'_y) induced by 3λ phonons with orbital indices $(m_x m_y)$, as calculated using the parameters listed in Supplementary Table 3. The phonon frequencies listed in the 2nd column were determined using the elastic properties of GaAs. Since the spacer of the MC includes (Al,Ga)As layers, these values sligthly underestimate the measured resonance frequencies. The corresponding frequencies for the λ phonons are three times as large. The energy splitting $\Delta E^{(p_x p_y)}$ between the states is give in terms of the trap asymmetry $\frac{\Delta a}{a}$ (cf. Eq. 24). The decay rate for all phonon and polariton modes in the cooperativity calculations were taken to be equal to the one of the ground state polariton and 3λ mode (see text for details).

INTRA-LEVEL COUPLING

mode	$f_{\rm BAW}$ (GHz)	$m_x m_y$	$p_x p_y$	ΔE (μeV)	$g^{p_x p_y}_{0,\uparrow\uparrow m_x m_y}$ (MHz)^a (MHz)^a	1st order: $g^{p_x p_y}_{0\uparrow\downarrow,m_x m_y}$ (MHz)^b	1st order: $1/C^{(max)}_{\uparrow\downarrow}$	2nd order: $G^{p_x p_y \leftrightarrow p'_x p'_y}_{2\uparrow\downarrow,m_x m_y}$ (MHz)$^{(c)}$	2nd order: $1/C^{(max)}_{2\uparrow\downarrow}$
TA	4.559	11	11	$-2.38\frac{\Delta a}{a}$	0	1.15	290	0.	∞
	4.559	11	12	$-2.11\frac{\Delta a}{a} - 1.59$	0	0.921	453	0.	∞
	4.559	11	22	$-2.38\frac{\Delta a}{a}$	0	0.737	708	0.	∞
LA'	6.496	11	11	$-2.38\frac{\Delta a}{a}$	6.68	$0.0285\frac{\Delta a}{a}$	$4.73\times10^5(\frac{\Delta a}{a})^{-2}$	$0.029\times10^{-3}\frac{\Delta a}{a}$	$1.13\times10^4(\frac{\Delta a}{a})^{-1}$
	6.496	11	12	$-2.11\frac{\Delta a}{a} - 1.59$	5.35	$0.0228\frac{\Delta a}{a}$	$7.39\times10^5(\frac{\Delta a}{a})^{-2}$	$0.019\times10^{-3}\frac{\Delta a}{a}$	$1.76\times10^4(\frac{\Delta a}{a})^{-1}$

INTER-LEVEL COUPLING

Mode	$f_{\rm BAW}$ (GHz)	$m_x m_y$	$p_x p_y$	$p'_x p'_y$	ΔE (μeV)	$g^{p_x p_y}_{0,m_x m_y}$ (MHz)^a	$g^{p'_x p'_y}_{0,m_x m_y}$ (MHz)^a	$g^{p_x p_y \leftrightarrow p'_x p'_y}_{0\uparrow\uparrow,m_x m_y}$ (MHz)^d	$1/C^{(max)}_{\uparrow\uparrow}$	$g^{p_x p_y \leftrightarrow p'_x p'_y}_{0\uparrow\downarrow,m_x m_y}$ (MHz)	$1/C^{(max)}_{2,\uparrow\downarrow}$
TA'	4.463	11	12	21	$2\times10^3\frac{\Delta a}{a}$	1.64	1.64	0.974	405	0.	∞
	4.336	22	21	12	$-2\times10^3\frac{\Delta a}{a}$	0.	0.	1.27	237	0.	∞
TA	4.559	11	12	21	$2\times10^3\frac{\Delta a}{a}$	0.	0.	$4.26\frac{\Delta a}{a}$	$21.2(\frac{\Delta a}{a})^{-2}$	0.	∞
	4.673	22	21	12	$-2\times10^3\frac{\Delta a}{a}$	0.	0.	0.	∞	0.755	675
LA	6.496	11	12	21	$2\times10^3\frac{\Delta a}{a}$	5.35	5.34	0.0593	1.09×10^5	0.	∞
	6.664	12	11	12	$-10^3\,(1+\frac{\Delta a}{a})$	0.	0.	6.1	∞	$0.102^{(f)}$	1.28×10^5
	6.815	22	21	12	$-2\times10^3\frac{\Delta a}{a}$	0.	0.	5.04	15.2	$0.076\frac{\Delta a}{a}$	$6.62\times10^4(\frac{\Delta a}{a})^{-1}$

(31)

[a] see Eq. 19.
[b] see Eq. 25.
[c] see Eq. 26.
[d] see Eq. 29.
[e] see Eq. 30.
[f] second-order spin-flip process.

D. Coupling strength and cooperativity

The upper and lower panels of Supplementary Table 5 summarized the coupling parameters related to phonon-mediated intra- and inter-level coupling of polariton levels, respectively. The table only includes data for optomechanical transitions with non-vanishing coupling strengths involving the TA', TA, and LA' phonon modes and polaritons with orbital indices m_i and p_i with $i = 1, 2$. The latter were determined using the parameters of Supplementary Table 3 for zero photon-exciton detuning, $\delta_{CX} = 0$. The phonon frequencies in the second column were obtained by the diagonalization of Eq. 8. The energy splitting between the states in the 5th column depends on the TE-TM splitting (cf. Eq. 24) and is stated as a function of the asymmetry $\frac{\Delta a}{a}$. For the pseudo-spin states, this splitting is typically of only a few μeV. The 7th and 8th columns list the level modulation strength $g^{p_x p_y}_{0,m_x m_y}$, which determines the strain-induced modulation amplitude of the level leading to emission sidebands. In agreement with the remarks in Sec. V C 2, the latter is only non-vanishing for the symmetric $(m_x m_y) = 1, 1$ phonons.

1. *Coupling and self-oscillations*

Optomechanical self-oscillations require phonon-induced transitions between polariton states spaced by one or two phonon energies, for first and second-order coupling processes, respectively. These transition energies ΔE between the involved states are listed in the 6th column of Supplementary Table 5. Only the $(m_x m_y) = (11)$ phonon can induce intra-level transitions between pseudo-spin states: ΔE for these transitions is normally small. Inter-level transitions can take place between between GS and ESs as well as between almost degenerated ESs. The former are always of second-order to due the large energy difference ($\Delta E \gg \Omega_{mec}$). The latter can be of first or second-order, depending

on the value of $\Delta a/a$. Note, however, that ΔE can also be significantly changed by a preferential population of one of the pseudo-spin levels via non-linear polariton-polariton interactions.

As a second requirement, self-oscillations pressuposes a sufficiently strong optomechanical coupling to overcome phonon and polariton decoherence. The latter can be quantified by the cooperativity $C^{(p_xp_y)}_{\uparrow\downarrow(\uparrow\downarrow)(m_xm_y)}$, an adimensional parameter giving the optomechanical interaction between phonons and polaritons with finite decoherence rates γ_{pol} and Γ_{mec}, respectively. For first-order processes, it is related to the coupling energy $g^{(p_xp_y)}_{(m_xm_y)}$ according to:

$$C^{(p_xp_y)\leftrightarrow(p'_xp'_y)}_{\uparrow\downarrow(m_xm_y)} = N_{pol}C^{(p_xp_y)\leftrightarrow(p'_xp'_y)}_{0,\uparrow\downarrow(m_xm_y)} \quad \text{with} \quad C^{(p_xp_y)\leftrightarrow(p'_xp'_y)}_{0,\uparrow\downarrow(m_xm_y)} = \frac{|g^{(p_xp_y)\leftrightarrow(p'_xp'_y)}_{0,\uparrow\downarrow(m_xm_y)}|^2}{4\gamma_{\mathrm{pol}}\Gamma_{\mathrm{mec}}} \quad \text{(1st order)}. \tag{32}$$

Here, N_{pol} is the polariton population and $C^{(p_xp_y)\leftrightarrow(p'_xp'_y)}_{0,\uparrow\downarrow(m_xm_y)}$ the single polariton cooperativity. Cooperativities exceeding unity mark the onset of self-oscillations between states split by one (or two, for second-order processes) phonon energies (see, e.g., Ref. [11]). For second-order processes, the cooperativity depends on the populations of the initial and final polariton states[12]. If one assumes both to be equal to N_{pol}, then one obtains the following expression for the second-order cooperativity:[12]

$$C^{(p_xp_y)\leftrightarrow(p'_xp'_y)}_{2,\uparrow\downarrow(m_xm_y)} = N_{pol}C^{(p_xp_y)\leftrightarrow(p'_xp'_y)}_{0,\uparrow\downarrow(m_xm_y)} \quad \text{with} \quad C^{(p_xp_y)\leftrightarrow(p'_xp'_y)}_{2,\uparrow\downarrow(m_xm_y)} = \frac{g^{p_xp_y\leftrightarrow p'_xp'_y}_{0,\uparrow\downarrow,m_xm_y}\, g^{(p_xp_y)}_{0,(m_xm_y)}}{4\Omega_{\mathrm{mec}}\Gamma_{\mathrm{mec}}} \quad \text{(2nd order)}. \tag{33}$$

Note that in contrast to the first-order, the second-order cooperativity does not depend of the polariton decoherence rate.

Columns 9 and 10 (11 and 12) in the upper part of Supplementary Table 5 list the coupling energies and associated inverse cooperativities for intra-level coupling of the first (second) order. In the calculations, we assumed that all involved polariton and phonon states have the same decay rate as the ones for the ground state listed in Supplementary Table 3. Following the definition, the inverse cooperativities yield the threshold polariton population for self-oscillations. Remarkably, the $(m_xm_y) = (11)$ TA mode requires less than 1000 polaritons for self-oscillations: this number is significantly smaller than the experimental values for the population in the condensation regime of 10^5 polaritons.

The spin-flip process involved in the self-oscillations is mediated by the component e^c_{xy} of the TA phonons (see, e.g., Supplementary Table 4). For LA' modes, this component depends on the trap asymmetry. Note, however, that LA' self-oscillations in traps with with $\Delta a/a < 1$ can also be triggered at polariton populations below the condensation threshold. We remind ourselves that condensation is nevertheless important to ensure long polariton coherences.

Low polariton population thresholds for self-oscillations are also supported by the results for inter-level coupling in the lower part of Supplementary Table 5. Except for the LA' transition ($m_xm_y\, p_xp_y\, p'_xp'_y = 12\,11\,12$), all relevant couplings are of first-order. Self-oscillations can be induced by both spin-conserving and spin-flitting inter-level transitions mediated by the shear as well as the uniaxial strain components of the phonon mode, respectively (see, e.g., Supplementary Table 4). As for intra-level coupling, multi-mode self oscillations can in both cases be triggered at polariton populations much less than the condensation threshold. Note, in addition, that spin-conserving processes mediated by the uniaxial components can yield record-high coupling strengths of several MHz, as indicated by the LA' ($m_xm_y, p_xp_y, p'_xp'_y = 12, 21, 12$) process.

The coupling energy calculations of Supplementary Table 5 can be easily extended to λ phonons. For the TA mode, the coupling energies do not depend on the phonon wavelength: the conclusions regarding self-oscillations also apply with minor corrections for the λ modes. In the case of the LA' mode, the spin-flipping coupling energy reduces by $(1/3)^2$ and the threshold densities increase by approximately one order of magnitude. The latter, however, may be partially compensated by the much longer lifetimes (and higher amplitudes) for the λ phonons (cf. Fig. 1).

E. Quadratic Hamiltonian and strong coupling

1. *Optomechanical parametric oscillator*

We write the Hamiltonian for two polaritonic modes coupled to a phonon as

$$\hat{H} = \hat{H}_0 + H_{\text{int}}, \tag{34}$$

$$\hat{H}_0 = \hbar\Omega_M \hat{b}^\dagger \hat{b} + \sum_{j=1,2} \hbar\omega_j \hat{a}_j^\dagger \hat{a}_j, \tag{35}$$

$$H_{\text{int}} = H_{\text{int},1} + H_{\text{int},2} + H_{\text{int},h}, \tag{36}$$

$$H_{\text{int},j} = \hbar g_j \hat{a}_j^\dagger \hat{a}_j \left(\hat{b}^\dagger + \hat{b}\right), \tag{37}$$

$$H_{\text{int},h} = \hbar g_h \left(\hat{a}_1^\dagger \hat{a}_2 + \hat{a}_2^\dagger \hat{a}_1\right)\left(\hat{b}^\dagger + \hat{b}\right) \tag{38}$$

where $H_{\text{int},1}$ and $H_{\text{int},2}$ couple the phonon with each mode separately whereas $H_{\text{int},h}$ involves a hopping term between the two modes. The strengths of these optomechanical coupling interactions are given by the rates g_1, g_2 and g_h. In these equations $\hat{a}_j^\dagger$ ($\hat{a}_j$) creates (annihilates) a polariton in the j-mode with energy $\hbar\omega_j$ and $\hat{b}^\dagger$ ($\hat{b}$) creates (annihilates) the cavity phonon having energy $\hbar\Omega_M$.

Our goal is to extract the most important mode mixing contribution of the optomechanical coupling H_{int} for the case in which the detuning between the two modes is two phonons, i.e., for $\omega_2 - \omega_1 = 2\Omega_M$. It is easy to see that the energy conserving process for mode mixing at such a detuning requires a second order process involving once $H_{\text{int},h}$ and once one of the $H_{\text{int},j}$ mode-conserving interactions: this, due to $j = 1, 2$ and ordering, means four different paths interfering. In order to obtain such a second order contribution we apply the unitary transformation $\widetilde{\hat{H}} = \exp(-\hat{S})\hat{H}\exp(\hat{S})$ and $\widetilde{\hat{H}} \approx \hat{H}_0 + \frac{1}{2}[\hat{H}_{\text{int}}, \hat{S}]$ provided that $[S, \hat{H}_0] = \hat{H}_{\text{int}}$. Using the energy eigenbasis of the unperturbed Hamiltonian, $\hat{H}_0|n_1, n_2, n_b\rangle = E_{n_1,n_2,n_b}|n_1, n_2, n_b\rangle$, the above commutator gives the required non-diagonal matrix elements of $\hat{S}$:

$$\langle n_1', n_2', n_b'|\hat{S}|n_1, n_2, n_b\rangle = \langle n_1', n_2', n_b'|\hat{H}_{\text{int}}|n_1, n_2, n_b\rangle/(E_{n_1,n_2,n_b} - E_{n_1',n_2',n_b'}). \tag{39}$$

For the detuning condition $\omega_2 - \omega_1 = 2\Omega_M$ the transformed Hamiltonian $\widetilde{\hat{H}}$ contains the following energy conserving non-diagonal term

$$\frac{(g_1 - g_2)g_h}{\Omega_M}\hat{a}_2^\dagger \hat{a}_1 \hat{b}^2 + h.c., \tag{40}$$

namely, a polariton transfer from the highest (lowest) energy mode to the lowest (highest) energy mode creates (destroys) two phonons. Thus this synthetic intermode coupling is quadratic in the phonon operators and has an effective coupling rate of $G_2 = (g_1 - g_2)g_h/\Omega_M$. In general g_1 is equal to g_2, however, different polariton populations of the modes can lead to $g_1 - g_2 \neq 0$ [13]. The above expression for G_2, assuming $g_\Delta = g_1 - g_2 = g_{0,\uparrow\uparrow}$, is used at the end of Sec. **Electrically stimulated phonoritons** of the main text.

2. *Quadratic Hamiltonian and RWA*

On the basis of measurements and arguments in Ref. [14], we assume that there exists an optomechanical coupling between two polaritonic modes involving a quadratic phonon operator. For generality, the modes are denoted l and u for the one of the lower energy and the higher energy one, respectively. Such interaction is included in the Hamiltonian

$$\hat{H} = \hbar\Omega_M \hat{b}^\dagger \hat{b} + \sum_{i=l,u} \hbar\omega_i \hat{a}_i^\dagger \hat{a}_i + \hbar G_2 \left(\hat{a}_u^\dagger \hat{a}_l + \hat{a}_l^\dagger \hat{a}_u\right)\left(\hat{b}^\dagger + \hat{b}\right)^2 + \hat{H}_{\text{driving}}. \tag{41}$$

For simplicity we assume a driving Hamiltonian that feeds independently and coherently each of the three modes via the rates β_l, β_u, and β_m with frequencies $\omega_{l,d}$, $\omega_{u,d}$, and Ω_d, respectively.

$$\hat{H}_{\text{driving}} = \hbar(\beta_m \hat{b} e^{i\Omega_d t} + \beta_l \hat{a}_l e^{i\omega_{l,d} t} + \beta_u \hat{a}_u e^{i\omega_{u,d} t} + h.c.) \tag{42}$$

However, we can also apply the results below to the case in which the polaritonic modes are nonresonantly driven. By moving to the interaction picture of

$$\hat{H}_0 = \hbar\Omega_d \hat{b}^\dagger \hat{b} + \sum_{i=l,u} \hbar\omega_{i,d} \hat{a}_i^\dagger \hat{a}_i, \tag{43}$$

fixing the condition $\omega_{u,d} - \omega_{l,d} = 2\Omega_d$, and discarding the fast oscillating terms we get an RWA Hamiltonian

$$\hat{H} = \hbar\Delta_m \hat{b}^\dagger \hat{b} + \sum_{i=l,u} \hbar\Delta_i \hat{a}_i^\dagger \hat{a}_i + \hbar G_2 \left(\hat{a}_u^\dagger \hat{a}_l \hat{b}^2 + \hat{a}_l^\dagger \hat{a}_u (\hat{b}^\dagger)^2 \right) + \hbar(\beta_m \hat{b} + \beta_u \hat{a}_u + \beta_l \hat{a}_l + h.c.) \tag{44}$$

with the detuning variables defined as $\Delta_m = \Omega_M - \Omega_d$ and $\Delta_i = \omega_i - \omega_{i,d}$, with $i = l, u$. Notice that within this RWA picture the choice of $\Delta_m = \Delta_l = \Delta_u = 0$ is the condition that we are interested in since this implies that $\omega_u - \omega_l = 2\Omega_M$, i.e., the detuning between the higher-energy state and the lower-energy one is resonant with the energy of two phonons.

3. *Strong coupling*

The optomechanical coupling of the Eq.(44) can be linearized considering fluctuations around the strong coherent amplitudes of each mode (arising due to the presence of polaritonic and mechanical driving by a BAWR transducer). First, following a similar procedure used to describe optomechanical strong-coupling for the case of linear optomechanical interaction in Ref. [11], we consider fluctuations in the upper-energy state which is *less* populated than the lower-energy one. For this we replace $\hat{a}_u \to \alpha_u + \delta\hat{a}_u$ and $\hat{a}_l \to \alpha_l$. We take the large numbers $\alpha_{l/u} = \sqrt{n_{l/u}}$ to be real and note that these strong coherent amplitudes of the polaritonic fields are set by the driving fields. Similarly, we write the mechanical mode operator as $\hat{b} = \alpha_b + \hat{\delta b}$ where $\hat{\delta b}$ is the mechanical fluctuation around the strong coherent amplitude of the mechanical field and take $\alpha_b = \sqrt{n_b}$ real. Indeed, for this detuning condition the system naturally tends to the large n_b regime even in absence of the phonon driving. It can be thought of as a result of polariton induced parametric driving of the phonon field, resembling a single mode squeezing that is limited by the nonlinearity. The amplitude of the mechanical RF driving, β_m, provides a knob to further increase n_b. Neglecting higher order terms and keeping the dominant interaction term for the case of strong mechanical driving, assuming $\sqrt{n_b} > \sqrt{n_u}$, we arrive to the following beam splitter Hamiltonian

$$\hat{H}_{m,u} = \hbar\Delta_m \hat{b}^\dagger \hat{b} + \hbar\Delta_u \hat{\delta a}_u^\dagger \hat{\delta a}_u + \hbar 2G_2\sqrt{n_b n_l} \left(\hat{\delta a}_u^\dagger \hat{\delta b} + \hat{\delta b}^\dagger \hat{\delta a}_u \right), \tag{45}$$

which describes the coherent energy exchange between the fluctuation of the mechanical field and the fluctuation of the polaritonic upper-energy state. Introducing the linewidth of the latter κ_u and the phonon linewidth Γ_m the equations of motion are

$$\frac{d}{dt}\begin{pmatrix} \langle \delta a_u \rangle \\ \langle \delta b \rangle \end{pmatrix} = -\mathrm{i} \begin{pmatrix} \Delta_u - \mathrm{i}\frac{\kappa_u}{2} & 2\sqrt{n_b n_l} G_2 \\ 2\sqrt{n_b n_l} G_2 & \Delta_m - \mathrm{i}\frac{\Gamma_m}{2} \end{pmatrix} \cdot \begin{pmatrix} \langle \delta a_u \rangle \\ \langle \delta b \rangle \end{pmatrix}. \tag{46}$$

Defining $g = 2\sqrt{n_b n_l} G_2$, $\delta = \Delta_u - \Delta_m$ and $\gamma = \Delta_u + \Delta_m$ the eigenfrequencies become

$$\omega_\pm = \frac{\gamma}{2} - \mathrm{i}\frac{\kappa_u + \Gamma_m}{4} \pm \sqrt{g^2 + \left(\frac{\delta + \mathrm{i}(\Gamma_m - \kappa_u)/2}{2} \right)^2} \tag{47}$$

For $\Gamma_m \ll \kappa_u$ and at resonance, $\Delta_u = \Delta_l = \Delta_m = 0$, the expression simplifies to

$$\omega_\pm = -\mathrm{i}\frac{\kappa_u}{4} \pm \sqrt{g^2 - \frac{\kappa_u^2}{4^2}} \tag{48}$$

and one can see that strong coupling arises when the effective interaction,

$$g = 2\sqrt{n_b n_2} G_2 > \kappa_u/4. \tag{49}$$

The imaginary part of $\omega_\pm$ being $\kappa_u/4$ implies that the peak linewidth is $\kappa_u/2$, i.e., a 50% reduction. This is due to the fact that these excitations are half mechanical and the phonon lifetime, being much larger than κ_u^{-1}, virtually does not contribute to the linewidth. Importantly, the compounded mechanical and optical enhancements of the effective coupling constant relaxes the number of phonons and polaritons required for reaching the strong coupling regime. The above procedure is similar to that describing strong coupling in the case of a linear optomechanical interaction as presented in Ref. [11].

We proceed further by noting that the optomechanical interaction of the RWA Hamiltonian can be written as $\hbar G_2(\hat{\Psi}^\dagger \hat{b}^2 + \hat{\Psi}(\hat{b}^\dagger)^2)$ with the operator $\hat{\Psi} = \hat{a}_l^\dagger \hat{a}_u$. It is easy to see that in absence of such interaction this polaritonic operator, decays with $(\kappa_u + \kappa_l)/2$ with κ_l the decay rate of the lower-energy state. Since $[\hat{\Psi}, \hat{\Psi}^\dagger] = \hat{a}_l^\dagger \hat{a}_l - \hat{a}_u^\dagger \hat{a}_u + 1$, it is convenient to define $\tilde{\hat{\Psi}} = \hat{\Psi}/\sqrt{n_l - n_u + 1}$, so that in the high occupation limit $[\tilde{\hat{\Psi}}, \tilde{\hat{\Psi}}^\dagger] = (\hat{a}_l^\dagger \hat{a}_l - \hat{a}_u^\dagger \hat{a}_u + 1)/(n_l -$

$n_u+1)=1$, i.e., this polaritonic excitation fulfills the bosonic commutation relation. Introducing this operator and the expansion $\hat{b}=\sqrt{n_b}+\hat{\delta b}$ in Eq.(44) we obtain a beam-splitter-like Hamiltonian

$$\frac{d}{dt}\begin{pmatrix}\langle\delta\widetilde{\Psi}\rangle\\ \langle\delta b\rangle\end{pmatrix}=-\mathrm{i}\begin{pmatrix}\Delta_u-\Delta_l-\mathrm{i}\frac{\kappa_u+\kappa_l}{2} & 2\sqrt{n_b(n_l-n_u+1)}G_2\\ 2\sqrt{n_b(n_l-n_u+1)}G_2 & \Delta_m-\mathrm{i}\frac{\Gamma_m}{2}\end{pmatrix}\cdot\begin{pmatrix}\langle\delta\widetilde{\Psi}\rangle\\ \langle\delta b\rangle\end{pmatrix}. \tag{50}$$

For $\Gamma_m\ll\kappa_u+\kappa_l$ and at resonance, $\Delta_u=\Delta_l=\Delta_m=0$, the eigenenergies simplifies to

$$\omega_\pm=-\mathrm{i}\frac{\kappa_u+\kappa_l}{4}\pm\sqrt{\tilde{g}^2-\frac{(\kappa_u+\kappa_l)^2}{4^2}} \tag{51}$$

For $n_l\gg n_u$, $\tilde{g}=2\sqrt{n_bn_l}G_2$ and one recovers the same optomechanical effective coupling as in Eq.49. For $\tilde{g}>(\kappa_u+\kappa_l)/4$ these solutions have linewidth $(\kappa_u+\kappa_l)/2$, i.e., half the original linewidth. Since the upper-energy state linewidth was shown to halve when $2\sqrt{n_bn_l}G_2\gg\kappa_u/4$, the halving of the sum of the linewidths indicates that the effective linewidth of the ground state also halves. We note that the operator $\hat{\widetilde{\Psi}}$ can be interpreted as describing a quasi-particle that represents the beating between states l and u, and which is resonantly coupled with the mechanics through the creation of two phonons.

Supplementary References

[1] A. S. Kuznetsov, D. H. O. Machado, K. Biermann, and P. V. Santos, Electrically Driven Microcavity Exciton-Polariton Optomechanics at 20 GHz, Phys. Rev. X **11**, 1 (2021).
[2] A. S. Kuznetsov, P. L. J. Helgers, K. Biermann, and P. V. Santos, Quantum confinement of exciton-polaritons in a structured (Al,Ga)As microcavity, Phys. Rev. B **97**, 195309 (2018).
[3] W. J. M. Naber, T. Fujisawa, H. W. Liu, and W. G. Van Der Wiel, Surface-acoustic-wave-induced transport in a double quantum dot, Phys. Rev. Lett. **96**, 136807 (2006).
[4] M. Metcalfe, S. M. Carr, A. Muller, G. S. Solomon, and J. Lawall, Resolved sideband emission of InAs/GaAs quantum dots strained by surface acoustic waves, Phys. Rev. Lett. **105**, 037401 (2010).
[5] M. Weiß, D. Wigger, M. Nägele, K. Müller, J. J. Finley, T. Kuhn, P. Machnikowski, and H. J. Krenner, Optomechanical wave mixing by a single quantum dot, Optica **8**, 291 (2021).
[6] G. Panzarini, L. C. Andreani, A. Armitage, D. Baxter, M. S. Skolnick, V. N. Astratov, J. S. Roberts, A. V. Kavokin, M. R. Vladimirova, and M. A. Kaliteevski, Exciton-light coupling in single and coupled semiconductor microcavities: Polariton dispersion and polarization splitting, Phys. Rev. B **59**, 5082 (1999).
[7] A. Kavokin, G. Malpuech, and M. Glazov, Optical spin hall effect, Phys. Rev. Lett. **95**, 136601 (2005).
[8] P. Yu and M. Cardona, *Fundamentals of Semiconductors: Physics and Materials Properties* (Springer, Heidelberg, 1995).
[9] D. Royer and E. Dieulesaint, *Elastic Waves in Solids I* (Springer, Berlin, 2000).
[10] G. L. Bir and G. E. Pikus, *Symmetry and Strain-Induced Effects in Semiconductors* (John Wiley & Sons, New York, 1974).
[11] M. Aspelmeyer, T. J. Kippenberg, and F. Marquardt, Cavity optomechanics, Rev. Mod. Phys. **86**, 1391 (2014).
[12] A. A. Reynoso, G. Usaj, D. L. Chafatinos, F. Mangussi, A. E. Bruchhausen, A. S. Kuznetsov, K. Biermann, P. V. Santos, and A. Fainstein, Optomechanical parametric oscillation of a quantum light-fluid lattice, Phys. Rev. B **in press**, 10.1103/PhysRevB.105.195310 (2022), arXiv:2112.15245v1 see also: https://physics.aps.org/articles/v15/s70.
[13] N. Carlon Zambon, Z. Denis, R. De Oliveira, S. Ravets, C. Ciuti, I. Favero, and J. Bloch, Enhanced cavity optomechanics with quantum-well exciton polaritons, Phys. Rev. Lett. **129**, 093603 (2022).
[14] D. L. Chafatinos, A. S. Kuznetsov, S. Anguiano, A. E. Bruchhausen, A. A. Reynoso, K. Biermann, P. V. Santos, and A. Fainstein, Polariton-Driven Phonon Laser, Nat. Commun. **11**, 1 (2020).
